# Sorcin regulate pyroptosis by interacting with NLRP3 inflammasomes to facilitate the progression of hepatocellular carcinoma

**DOI:** 10.1038/s41419-023-06096-1

**Published:** 2023-10-13

**Authors:** Zhenfen Li, Ziyue Yang, Yuanyuan Zhu, Chunmeng Fu, Ning Li, Fang Peng

**Affiliations:** 1 Department of Blood Transfusion, Clinical Transfusion Research Center, Xiangya Hospital, Central South University, Changsha, Hunan China; 2 National Health Commission (NHC) Key Laboratory of Cancer Proteomics, Xiangya Hospital, Central South University, Changsha, Hunan China; 3 National Clinical Research Center for Geriatric Disorders, Xiangya Hospital, Central South University, Changsha, China

**Keywords:** Liver cancer, Metastasis, Tumour biomarkers

## Abstract

A high recurrence rate and easy metastasis are two prominent clinical features of hepatocellular carcinoma (HCC), which is also the most common cause of cancer-related death. However, the molecular pathogenesis of HCC remains unclear. Soluble resistance-related calcium-binding protein (Sorcin) is highly expressed in a variety of tumor cell lines and multidrug-resistant cell lines and participates in the malignant progression of tumors by regulating apoptosis. Pyroptosis is also a form of programmed cell death that plays a crucial role in exerting tumor suppression function and evoking anti-tumor immune responses. However, there is no consensus that Sorcin promotes HCC progression by regulating pyroptosis. Our study manifested that Sorcin was considerably upregulated, whereas pyroptosis-associated proteins were significantly decreased in HCC tissues and cells. Sorcin silencing attenuated the proliferation, migration, and invasion of HCC cells. Knockdown of Sorcin activates pyroptosis, and overexpression of Sorcin inhibits pyroptosis, yet has no significant effect on apoptosis, ferroptosis, and autophagy in HCC cells. Furthermore, coimmunoprecipitation and immunofluorescence assays revealed that Sorcin interacted with NLRP3 inflammasome to regulate pyroptosis in HCC cells. Then, the NLRP3 inhibitor MCC950 inhibited the activation of Sorcin knockdown-induced pyroptosis and reversed the effect of Sorcin silencing-induced weakening of malignant biological behavior in HCC. Similarly, suppression of Caspase-1 reversed the inhibitory effect of Sorcin knockdown on the malignant progression of HCC via knockdown of Caspase-1 or the inhibitor VX765. Consistent with the in vitro results, the nude mouse experiment showed that Sorcin knockdown inhibited the growth of HCC by activating pyroptosis, while Caspase-1 knockdown partially restored the growth inhibition caused by Sorcin knockdown. Collectively, high Sorcin expression in HCC negatively regulates pyroptosis by interacting with the NLRP3 inflammasome to promote HCC proliferation, migration, and invasion. The results of this study provide a scientific basis for Sorcin as a new biomarker and potential therapeutic target for HCC.

## Introduction

Liver cancer is a common malignant tumor with a high mortality rate worldwide [1], and approximately 90% of primary liver cancer is hepatocellular carcinoma (HCC) [2]. A high recurrence rate and easy metastasis are vital clinical characteristics of HCC, approximately 40–70% of patients with surgical resection relapse within 5 years [3, 4]. The therapeutic effect of HCC patients remains limited due to drug resistance and frequent recurrence and metastasis, leading to poor prognosis of HCC patients, which is also the main cause of death in HCC patients. Therefore, it is particularly important to explore novel therapeutic targets and molecular mechanisms to provide clinical guidance for the treatment and prognosis of HCC.

Soluble resistance-related calcium-binding protein (Sorcin) is a cytosolic protein prevalent in both normal and tumor cells previously designated V19. SRI is the gene encoding the protein (Gene ID:6717) [5]. As a calcium-binding protein, Sorcin is involved in tumor progression and maintenance of tumor multidrug resistance phenotypes by altering intracellular Ca^2+^ concentration, affecting signaling transduction, and altering the activity and function of other Ca^2+^-regulated proteins [6–8]. Sorcin is also highly expressed in tumor cell lines and multidrug-resistant cell lines and is closely associated with the malignancy and poor prognosis of various tumors [9–15]. Knockdown Sorcin inhibited epithelial-mesenchymal transitions of tumor cells by regulating the expression of E-cadherin, thereby inhibiting breast cancer cell metastasis [16]. Overexpression of Sorcin promotes colon cancer invasion and metastasis by activating the PI3K/Akt signaling pathway [14]. Additionally, Sorcin could regulate the apoptosis pathway to promote tumor progression, and overexpression of Sorcin leads to a significant decrease in cytosolic calcium levels and enhanced cell apoptosis resistance, which could prevent endoplasmic reticulum stress and escape apoptosis caused by chemotherapy drugs [17] while silencing Sorcin leads to increased levels of pro-apoptotic genes and induction of mitochondrial apoptotic pathways in cancer [18]. Therefore, Sorcin plays an important role in tumor progression and has the potential to become a novel biomarker and clinical target for tumor prevention and treatment. However, the role and molecular mechanism of Sorcin in HCC development remains unclear.

Pyroptosis, also known as cellular inflammatory necrosis, is a newly discovered form of programmed cell death (PCD) [19, 20]. It is characterized by continuous cell expansion until the cell membrane ruptures, leading to the release of cellular contents and thus activating a powerful inflammatory response [21]. NLRP3 acts as a sensor and mediates self-oligomerization to recruit an adaptor ASC [22]. Subsequently, the assembled ASC recruits an effector Caspase-1 through isotypic CARD–CARD domain interactions to form the NLRP3–ASC–Caspase-1 protein complex, called the NLRP3 inflammasome [23, 24]. The Caspase-1 pathway senses endogenous danger signals or environmental irritants through inflammasomes and recruits and activates Caspase-1, which cleaves and activates IL-18, IL-1β, and other inflammatory factors, cleaves the N-terminal sequence of GSDMD and makes it bind to the membrane to produce membrane pores, leading to cell pyroptosis [25–27]. As a form of inflammatory cell death, pyroptosis plays a critical role in tumor suppression by stimulating the anti-tumor immune response. Therefore, inducing pyroptosis in tumor cells is a potential new strategy for cancer therapy [28, 29]. When pyroptosis occurs, tumor cells can release tumor antigens and damage-associated molecular patterns to initiate adaptive immunity, thereby increasing the efficiency of immune checkpoint blockade to cause tumor regression, while the body can generate immune memory to prevent tumor recurrence [30, 31].

Our findings indicated that through interaction with NLRP3 inflammasome, Sorcin inhibited the assembly and activation of the NLRP3 inflammasome, further inhibiting cell activation and promoting HCC progression in this study. These findings provide a new biomarker and potential clinical therapeutic target for early diagnosis, metastasis, and prognosis evaluation of HCC.

## Materials and methods

### Cell culture

We obtained HCC cells (HuH7 and HCC-LM3), the immortalized hepatocyte cell line L02, and 293T cells from the National Health Commission Key Laboratory of Cancer Proteomics, Xiangya Hospital (Changsha, China). The cells were cultured in Dulbecco’s modified Eagle medium (DMEM, Gibco, USA) containing 10% fetal bovine serum (FBS, NEWZERUM, New Zealand) and 100 U/mL penicillin and streptomycin in a humidified incubator at 37 °C and 5% CO2. All cell lines were tested for the absence of mycoplasma contamination.

### Lentivirus production and transfection

Lentiviral plasmids containing pMD2. G, psPAX2, and target genes or corresponding control lentiviral plasmids (Supplemental Table S1) were cotransfected into 293T cells using polyethyleneimine linear according to the manufacturer’s instructions to produce lentiviral particles. Viral supernatant collection at 48 h and 72 h was performed after transfection. The virus supernatants were added to HCC cells, and the transduced cells were screened with puromycin or hygromycin B 72 h after infection.

### RNA extraction and qPCR

According to the manufacturer’s instructions, total RNA was extracted from the cell lines using TRIzol reagent (Invitrogen, USA). We assessed the quality of the sample with a NanoDrop^TM^ 1000. A TaqMan MicroRNA Reverse Transcription Kit was used to synthesize complementary DNA. qRT‒PCR was performed using the miScript SYBR Green PCR kit (Qiagen) on an ABI7500 instrument (Applied Biosystems). The endogenous control for RNA quantification was GAPDH. The target gene primers were listed in Supplemental Table S2.

### Immunohistochemistry

A total of thirty HCC samples and adjacent nontumorous tissue from HCC patients undergoing surgical resection were collected at the Xiangya Hospital of Central South University, Changsha, China. Immunohistochemical staining was performed following the protocol provided in the kit (ZSGB-BIO, PV-9000). The primary antibodies used are shown in supplementary Table S3. The study was approved by Xiangya Hospital’s Medical Ethics Committee.

### Western blot and antibodies

Cells were collected and lysed with RIPA buffer (RIPA, Beyotime, China). Samples of cell extract were combined with 5×SDS-reducing sample buffer and boiled for 10 min before loading. Proteins from each sample were separated using 10% SDS‒PAGE and then transferred onto a PVDF membrane. The membranes were blocked with 5% bovine serum albumin for 1 h and incubated overnight with primary antibodies at 4 °C. Subsequently, the membranes were incubated for 2 h with the corresponding secondary antibodies, and immunoreactive bands were visualized with a chemiluminescence system and quantified using ImageJ software. The working dilutions of primary antibodies are shown in Supplementary Table S3. The full and uncropped western blot involved in this study is presented as Supplementary Material.

### Coimmunoprecipitation

HCC cell samples, lysed with coimmunoprecipitation lysis buffer, were incubated with specific antibodies or the same species of IgG overnight at 4 °C. Then, protein A/G magnetic beads were added again for 2 ~ 3 h incubation. The supernatant of lysed 293T cell samples was incubated with only anti-Flag magnetic beads for 2 ~ 3 h at 4 °C. Subsequently, protein complexes were extensively washed with RIPA buffer. Finally, the precipitated proteins were eluted by boiling them in 2×SDS sample buffer and subjected to SDS‒PAGE analysis.

### Immunofluorescence staining

After 24 h of cell climbing, 4% paraformaldehyde was fixed for 30 min, 0.25% Triton X-100 was ruptured for 40 min, and 5% bovine serum albumin was blocked for 1 h. Incubation with primary antibodies at 4 °C overnight was followed by incubation with fluorescent secondary antibodies for 1.5 h. Staining the nuclei before capturing images was performed using DAPI. A confocal microscope (Zeiss, Oberkochen, Germany) was used to acquire the images.

### Cell viability assay

To assess cell proliferation, transfected cells were seeded at a density of 1000 cells per well in 96-well plates. We evaluated the viability of cells at 0, 1, 3, and 5 days after seeding using the Cell Counting Kit-8 (CCK-8) system. A CCK-8 solution of 10 μL was added to each well of the plate and incubated in darkness for 2 h at 37 °C. The proliferation status of cells could be reflected by absorbance at 450 nm using a microplate reader (BioTek, USA) at respective time points. Three technical repetitions were performed.

### Wound-healing

When cell growth fusion was up to 100%, with a sterile 10 μL pipette tip, we created a scratch wound and washed it to remove floating cells. Following scratching, the supernatant was replaced with serum-free medium, and photographs were taken under an inverted microscope at 0 h, 24 h, and 48 h intervals. Wound size was quantified by ImageJ. The wound-healing percentage was calculated and analyzed.

### Transwell assay

Cells were seeded into the upper chamber of transwell 24-well plates at a density of 2 × 10^^5^ in 200 μL of serum-free medium for migration analysis or chambers pretreated with Matrigel (Corning, 356234) for invasion analysis. Then, 500 μL culture medium with 10% FBS was added to the bottom of the chambers. After incubation for 24 h, migrated cells on the lower surface were fixed with 4% paraformaldehyde for 30 min and stained with 0.1% crystal violet stain solution (Solarbio, G1063) for 20 min. Subsequently, the migrated cells on the lower membrane surface were photographed and counted under the microscope.

### Mice

Eighteen BALB/c nude mice aged four to five-week-old were purchased and maintained in the specific pathogen-free animal experiment center of Hunan Yuan Tai Biotechnology Co., Ltd. (Hunan, China). BALB/c nude mice were divided randomly into three groups. HCC-LM3 cells (4.8 × 10^6^) were injected subcutaneously into the armpit of nude mice in each group, and the tumor size measurement was performed every 2-3 days. Tumor volume was calculated using the following formula: V = L × W^2^ × 1/2 (V, volume; L, length; W, width). At the end of the experiment, the mice were euthanized. The tumor tissues were assessed and analyzed by immunohistochemistry staining.

### Statistical analysis

GraphPad Prism software (version 9.0) was used for all analyses, and the results are presented as the mean±SD of at least three independent experiments. Statistical differences between the two groups were evaluated using a two-tailed Student’s unpaired t-test, and multiple comparisons were performed using one-way ANOVA.

## Results

### Sorcin is highly expressed in HCC

**Fig. 1:**
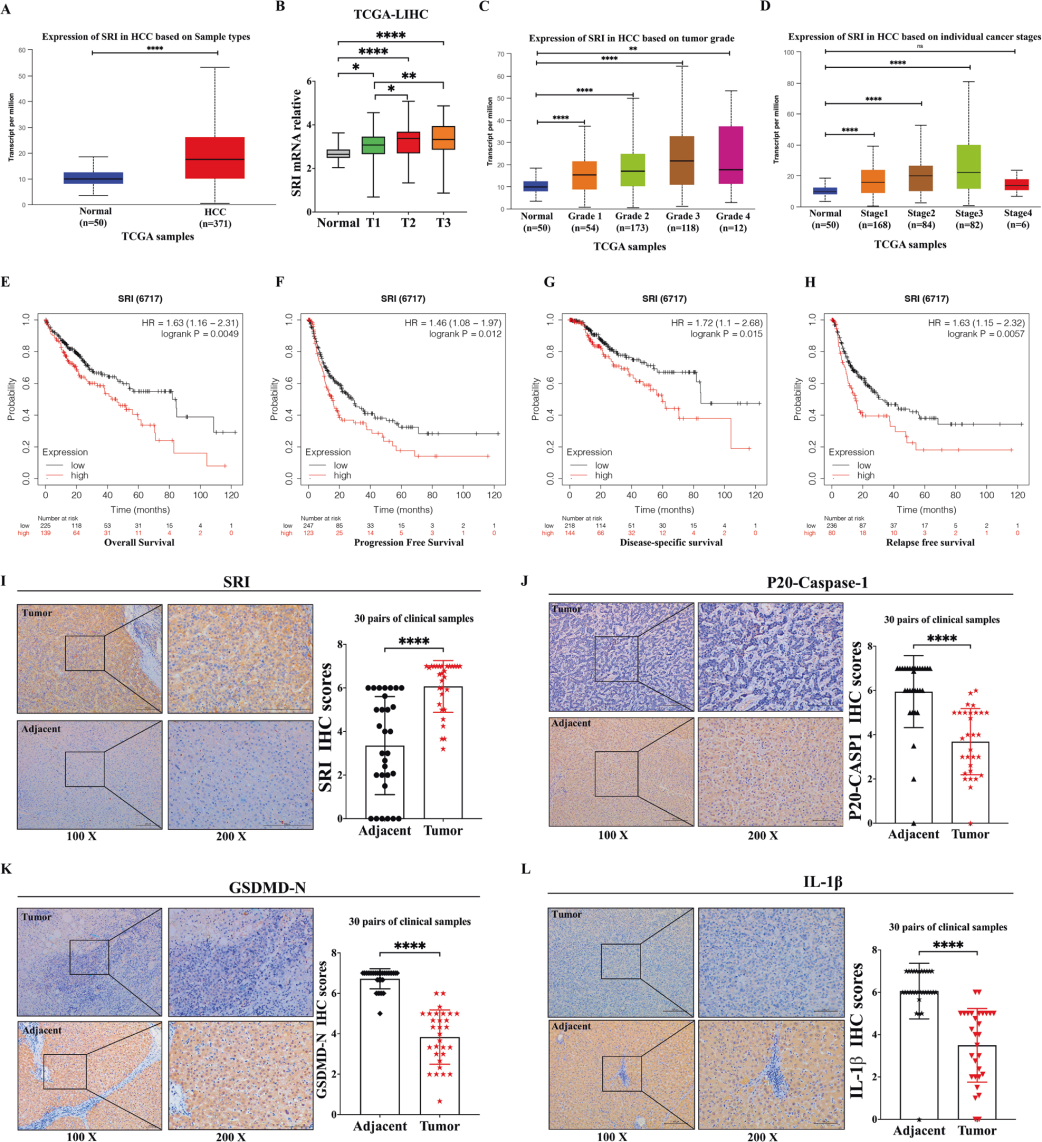
Sorcin expression, clinicopathological characteristics, and prognostic value in hepatocellular carcinoma (HCC). **A** Sorcin expression is higher in HCC (*n* = 371) than in normal liver tissues (*n* = 50) via the UALCAN database. **B** Sorcin expression was significantly associated with the advanced tumor-node-metastasis (TNM) stage. **C** The correlation between the transcriptional level of Sorcin and different tumor grades in HCC via the UALCAN database. **D** The association between Sorcin transcriptional level and tumor stages in HCC via the UALCAN database. **E**–**H** A Kaplan–Meier analysis of overall survival, progression-free survival, disease-specific survival, and relapse-free survival in HCC patients with high-Sorcin and low-Sorcin expression. **I** Immunohistochemical staining of Sorcin expression level was statistically higher in HCC tissues compared to adjacent tissues, and the staining results were statistically significant (*n* = 30). **J**–**L** Immunohistochemical staining of the pyroptosis-associated proteins was stained more lightly in HCC tissues than in adjacent tissues, including P20-Caspase-1, GSDMD-N, and IL-1β, and the difference was statistically significant. All ^*^P < 0.05, ^**^P < 0.01, ^****^P < 0.0001, and ns no significance.

Pan-cancer analysis showed that Sorcin was significantly higher in 16 tumors than in the corresponding paracancerous tissues (Supplementary Fig. S1). Transcriptome data of the HCC cohort indicated that Sorcin was markedly increased in HCC samples compared to the corresponding normal tissue samples (Fig. 1A). Furthermore, upregulated Sorcin expression was significantly associated with advanced tumor-node-metastasis stage, and higher Sorcin expression was associated with higher pathological grade and clinical stage in HCC patients (Fig. 1B–D). Additionally, HCC patients with higher Sorcin expression had a worse prognosis than those with lower expression (Fig. 1E–H).

### Sorcin facilitates the metastasis and proliferation of HCC cells

**Fig. 2:**
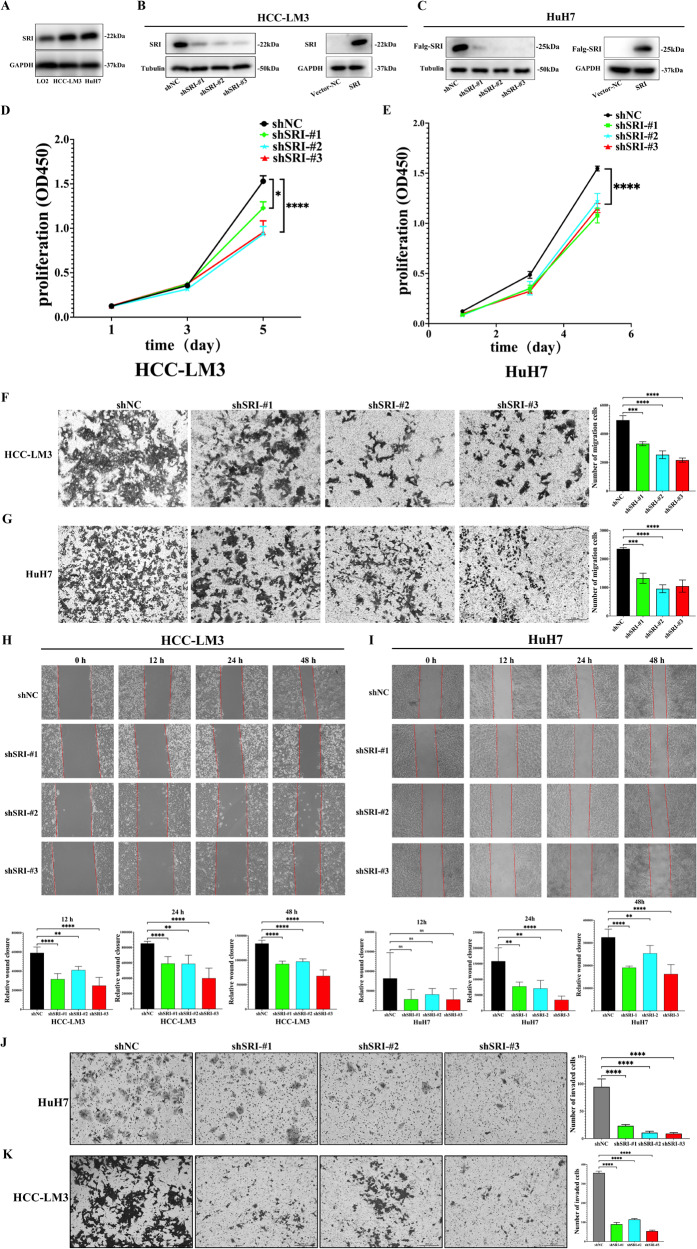
Knockdown Sorcin inhibited HCC cell proliferation, migration, and invasion. **A** Sorcin expression was higher in HCC-LM3/Huh-7 cells than in immortalized normal liver cells L02 by western blot. **B**, **C** Knockdown and overexpression of Sorcin were achieved in HCC-LM3 and HuH7 cells. **D**, **E** The CCK8 assays showed that knockdown Sorcin suppressed the proliferation of HCC-LM3/HuH7 cells. **F**, **G** The transwell assays accessed the effects of Sorcin knockdown on cell migration in HCC-LM3 and HuH7 cells. **H**, **I** Wound-healing assays, representative images of the HCC-LM3/HuH7 cells showed Sorcin downregulation inhibited its migration after scratching 0 h, 12 h, 24 h, and 48 h. **J**, **K** The transwell invasion assays to analyze the effect of Sorcin knockdown on the invasion ability of HCC-LM3 and HuH7 cells. Data are indicated as mean±SD; multiple comparisons were performed using one-way ANOVA; ^*^P < 0.05, ^**^P < 0.01, ^***^P < 0.001, ^****^P < 0.0001, and ns no significance.

Immunohistochemistry showed that compared with the adjacent normal tissues, Sorcin was highly expressed while P20-Caspase-1, GSDMD-N, and IL-1β were lowly expressed in HCC tissues (Fig. 1I–L). Western blot results showed that Sorcin in HCC cell lines (HCC-LM3, HuH7) was significantly higher than that in immortalized normal liver cells (L02) (Fig. 2A).

To verify the effect of Sorcin on the biological behavior of HCC, Sorcin knockdown was achieved in HCC-LM3 and HuH7 cells (Fig. 2B, C, supplementary Fig. S2). The CCK-8 tests demonstrated that Sorcin knockdown could decrease the cell proliferation ability of HCC cells when compared to the control group (25.38%, 40.06%, and 42.82% reduction in HCC-LM3 cells, 33.25%, 24.27%, and 25.23% reduction in HuH7 cells, respectively) (Fig. 2D, E). Wound-healing and transwell migration assays indicated that Sorcin knockdown significantly inhibited cell migration in HCC cells. Control cells had significantly more wound healing area than Sorcin knockdown cells by 48 h in wound healing assays (Fig. 2H, I). Transwell migration assays showed that Sorcin depletion severely reduced the migration ability of HCC cells in vitro by 27.59%, 41.82%, and 49.25% in HCC-LM3 cells, and by 35.75%, 46.58%, and 56.09% in HuH7 cells, as compared to control cells, respectively (Fig. 2F, G). Additionally, transwell invasion assays confirmed that Sorcin knockdown potently suppressed cell invasion in HCC cells (74.77%, 67.56%, and 84.47% reduction in HCC-LM3 cells, 50.39%, 74.42%, and 66.67% reduction in HuH7 cells, compared with the control group, respectively (Fig. 2J, K). These results showed that Sorcin promoted the development of HCC by promoting migration, invasion, and proliferation of HCC cells.

### Sorcin regulates pyroptosis to promote HCC progression

To further explore the molecular mechanism of Sorcin in HCC progression, we performed GO and KEGG pathway enrichment analyses on Sorcin. The results showed that Sorcin played a significant role in diverse biological processes and molecular functions, including a death−inducing signaling complex, and death receptor binding, and was enriched in multiple signal panels, such as apoptosis, Notch, and TNF signaling pathway (Supplementary Fig. S3). These results suggested that Sorcin may be involved in the regulation of cell death.

**Fig. 3:**
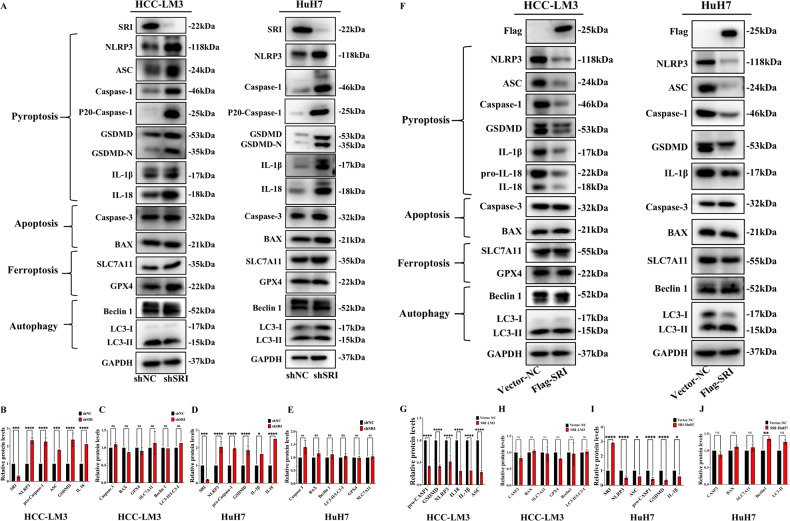
Sorcin regulates pyroptosis to promote HCC progression. **A** Knockdown Sorcin increased pyroptosis-related proteins, whereas apoptosis-, ferroptosis-, and autophagy-related proteins were not significantly different from those in the control group at the protein level in HCC-LM3 and HuH7 cells. **B**–**E** The bar graph shows the relative protein levels of pyroptosis-, apoptosis-, ferroptosis-, and autophagy-related proteins from three independent experiments by western blot. **F**–**J** Overexpression of Sorcin reduced the expression of the pyroptosis-related proteins in HCC-LM3 and HuH7 cells. Data are indicated as mean±SD; multiple comparisons were performed using Two-Way ANOVA; ^*^P < 0.05, ^**^P < 0.01, ^***^P < 0.001, ^****^P < 0.0001, and ns no significance.

Then, western blot was used to detect the expression of PCD-related proteins in Sorcin-knockdown and Sorcin-overexpressing HCC cell lines. Knockdown Sorcin increased the expression of pyroptosis-related proteins, including NLRP3, ASC, Caspase-1, P20-Caspase-1, GSDMD, GSDMD-N, IL-18, and IL-1β. Other PCD-related proteins were not significantly different from those in the control group, including apoptosis-related proteins (Caspase-3, BAX), ferroptosis-related proteins (SLC7A11, GPX4), and autophagy-related proteins (LC3 II/I, Beclin 1) (Fig. 3A–E). The results suggested that knockdown Sorcin promoted pyroptosis in HCC cells but had no significant effect on apoptosis, ferroptosis, or autophagy. Conversely, compared with the control group, the Sorcin overexpression groups (Flag-SRI) had decreased expression of pyroptosis-related proteins. Nevertheless, there was no significant difference in the expression levels of Caspase-3, BAX, SLC7A11, GPX4, LC3 II/I, and Beclin 1 (Fig. 3F–J).

### Sorcin interacted with NLRP3 inflammasome to regulate pyroptosis

**Fig. 4:**
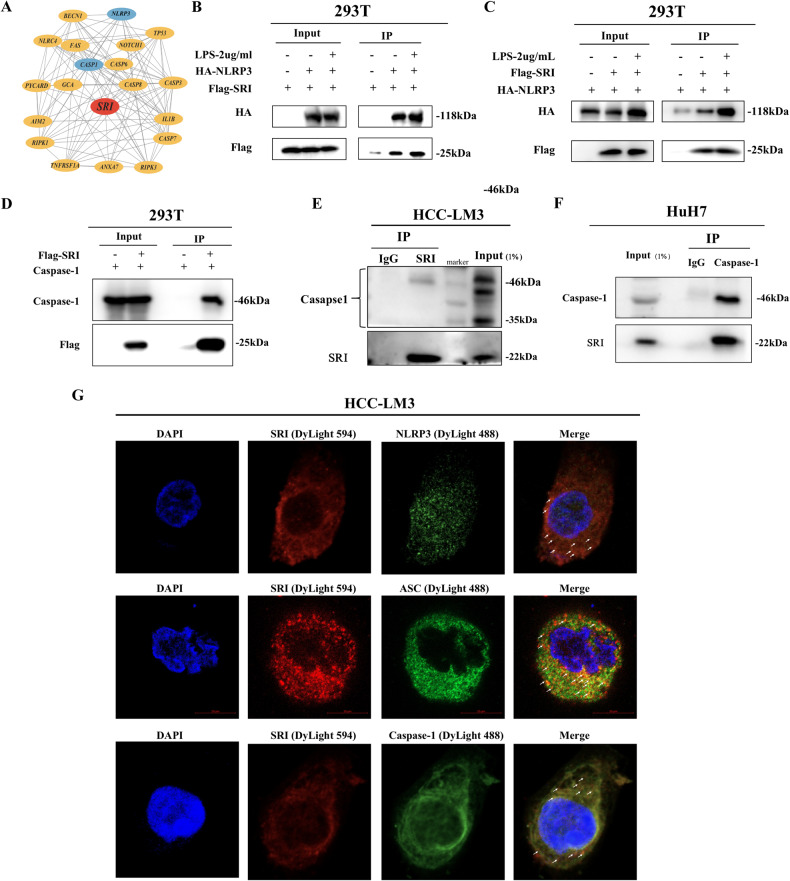
Sorcin interacted with NLRP3 inflammasomes. **A** Protein-protein interaction network of Sorcin and its interaction proteins according to the data from the FpClass database. **B**, **C** Exogenous co-immunoprecipitation assay showed the interaction between NLRP3 and Sorcin in 293T cells. **D** Exogenous Caspase-1 and Sorcin interacted in 293T cells as demonstrated by co-immunoprecipitation assays. **E** Co-immunoprecipitation assay showed the interaction between Sorcin and Caspase-1 in HCC-LM3 cells. **F** Caspase-1 interact with endogenous Sorcin in Huh7 cells by co-immunoprecipitation assay. **G** Cellular immunofluorescence proved that NLRP3 inflammasome and Sorcin are co-localized in the cytoplasm in HCC-LM3 cells.

To further investigate the specific molecular mechanisms by which Sorcin regulates HCC cell pyroptosis, the predicted Sorcin and its interacting protein data were downloaded from the FpClass database, and then protein-protein interaction networks were constructed using Cytoscape software. Sorcin may interact with NLRP3 and Caspase-1 (Fig. 4A).

Next, the coimmunoprecipitation-western blot results showed that firstly Sorcin interacted with NLRP3 inflammasome proteins (NLRP3 and Caspase-1) in 293T cells, further verifying an endogenous interaction between Caspase-1 and Sorcin in HuH7 and HCC-LM3 cells (Fig. 4B–F). Furthermore, immunofluorescence showed the co-localization between Sorcin and the NLRP3 inflammasome (NLRP3, ASC, and Caspase-1) in HCC-LM3 cells (Fig. 4G). These results suggested that Sorcin interacted with NLRP3 inflammasome in HCC cells.

In addition, Sorcin negatively regulated the expression of NLRP3 and Caspase-1 in HCC cell lines with Sorcin knockdown and overexpression (Fig. S5).

### Sorcin regulates NLRP3 inflammasome-mediated pyroptosis to promote HCC progression

**Fig. 5:**
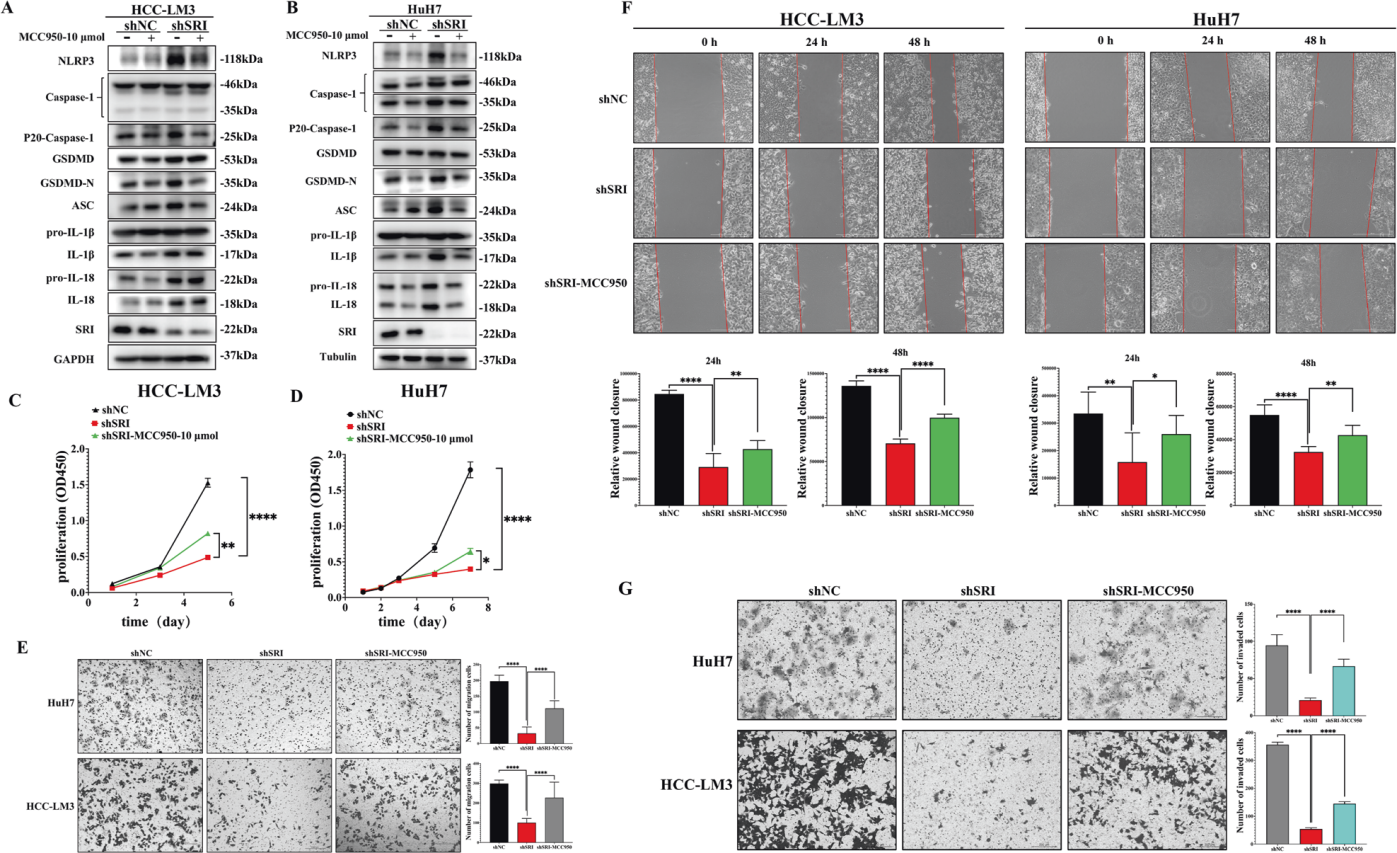
MCC950 reverses the inhibitory effect of Sorcin knockdown on the proliferation, migration, and invasion ability of HCC cells. **A**, **B** HCC-LM3 and HuH7 cells of Sorcin knockdown with shRNA interference were pre-treated with 10 μmol MCC950 for 24 h. Western blot results demonstrated that by blocking NLRP3 with MCC950, Sorcin inhibition-induced upregulation of Caspase-1, GSDMD, IL-1β, and IL-18 proteins was notably inhibited in HCC-LM3 and HuH7 cells. **C**, **D** The CCK8 assays showed that knockdown Sorcin suppressed the proliferation of HCC-LM3/HuH7 cells, while MCC950 partially restored the inhibitory effect of Sorcin knockdown on the proliferation capacity of HCC cells. **E** Transwell assays revealed that NLRP3 inhibition by MCC950 promoted cell migration in HCC cell lines. **F** Cell wound-healing proved that suppression of NLRP3 by MCC950 enhanced the ability of cell migration in HCC-LM3 and HuH7 cells at 0 h, 24 h, and 48 h. **J** The transwell invasion assays manifested that NLRP3 inhibitor MCC950 can reverse the inhibitory effect of Sorcin knockdown on the invasion ability of HCC cells. All: ^*^P < 0.05, ^**^P < 0.01, ^****^P < 0.0001, and ns no significance.

Previous studies showed that NLRP3 cleaves and activates Caspase-1 to trigger pyroptosis, and NLRP3 inflammasome is composed of NLRP3, ASC, and Caspase-1. MCC950, a selective NLRP3 inhibitor, blocks canonical and noncanonical NLRP3 activation. Western blot results further revealed that by blocking NLRP3 with MCC950, Sorcin inhibition-induced upregulation of Caspase-1, GSDMD, IL-1β, and IL-18 proteins was notably inhibited in HCC-LM3 and HuH7 cells (Fig. 5A, B). The CCK-8 assays manifested that Sorcin-silenced markedly inhibited the proliferation ability of HCC cells, while this inhibitory effect was reversed by MCC950 (Fig. 5C, D). Wound-healing and transwell assays revealed that MCC950 reversed the inhibitory effect of Sorcin knockdown on the migration and invasion ability of HCC cells (Fig. 5E–J). Therefore, Sorcin inhibition-elicited repression of proliferation, migration, and invasion, and activation of pyroptosis in HCC cells were mediated by NLRP3.

### Inhibition of Caspase-1 reversed the inhibitory effect of Sorcin knockdown on the malignant progression of HCC

**Fig. 6:**
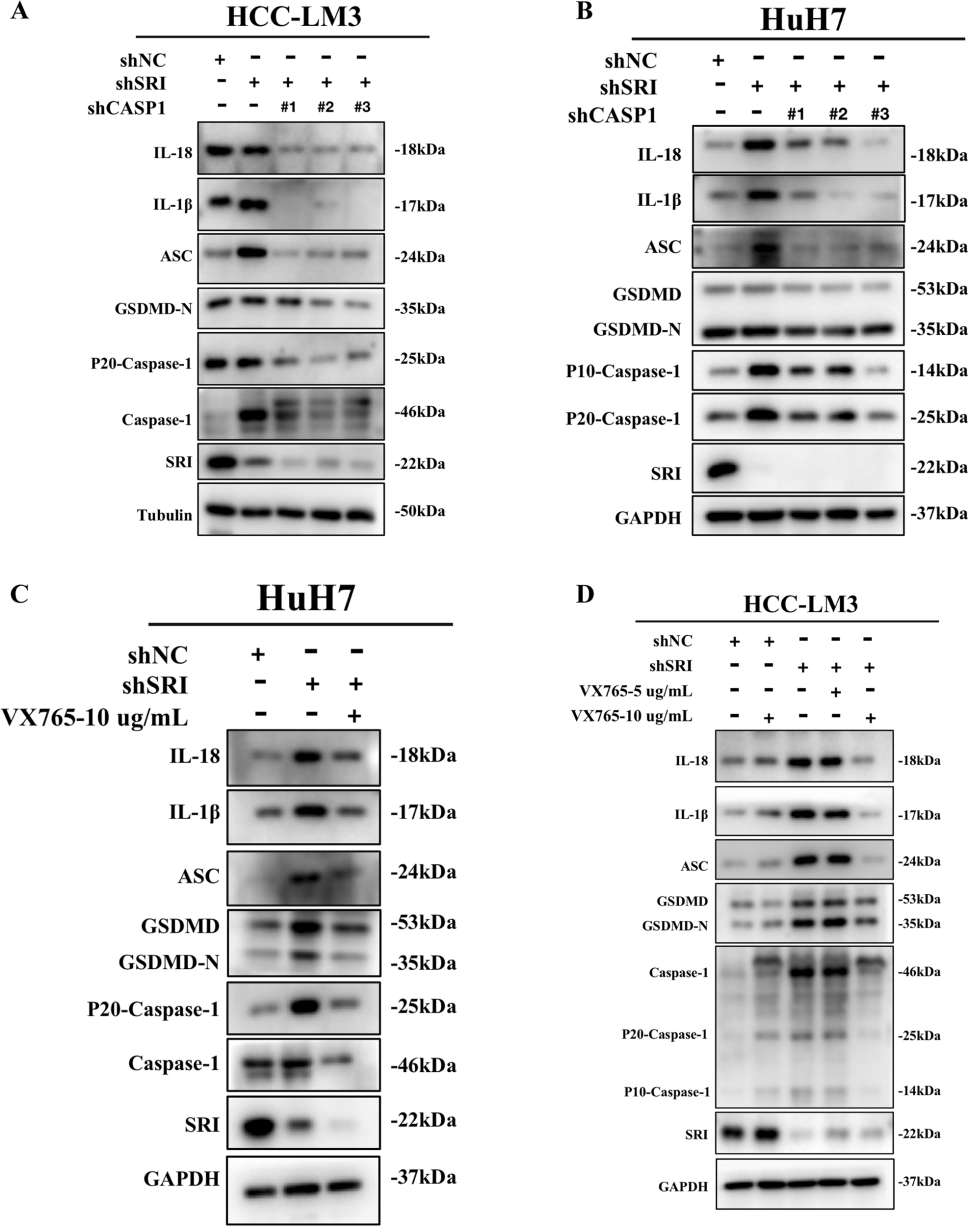
Suppression of Caspase-1 expression inhibited the activation of pyroptosis caused by Sorcin knockdown. **A**, **B** Western blot analysis showed that Caspase-1 knockdown reduced the upregulation of GSDMD-N, IL-1β, and IL-18 caused by Sorcin-silenced in HCC-LM3 and HuH7 cells. **C**, **D** HCC-LM3 and HuH7 cells were pre-treated with 5 μg/mL or 10 μg/mL VX765 for 24 h, and then protein levels of pyroptosis-related proteins were measured by western blot and normalized to GAPDH.

We constructed HCC cell lines with simultaneous knockdown of Sorcin and Caspase-1 (shSRI-shCASP1-LM3, shSRI-shCASP1-HuH7). Western blot results revealed that Sorcin-silenced increased the upregulation of pyroptosis-related proteins, while knockdown Caspase-1 reduced the upregulation of GSDMD-N, IL-1β, and IL-18 caused by Sorcin silencing in HCC cells (Fig. 6A, B). Belnacasan (VX-765), a specific Caspase-1 inhibitor, was also employed in our study. The VX765 inhibitor reversed the upregulation of pyroptosis-related proteins caused by Sorcin knockdown (Fig. 6C, D). Therefore, suppression of the expression of Caspase-1 inhibited the activation of Caspase-1-dependent pyroptosis induced by Sorcin knockdown.

**Fig. 7:**
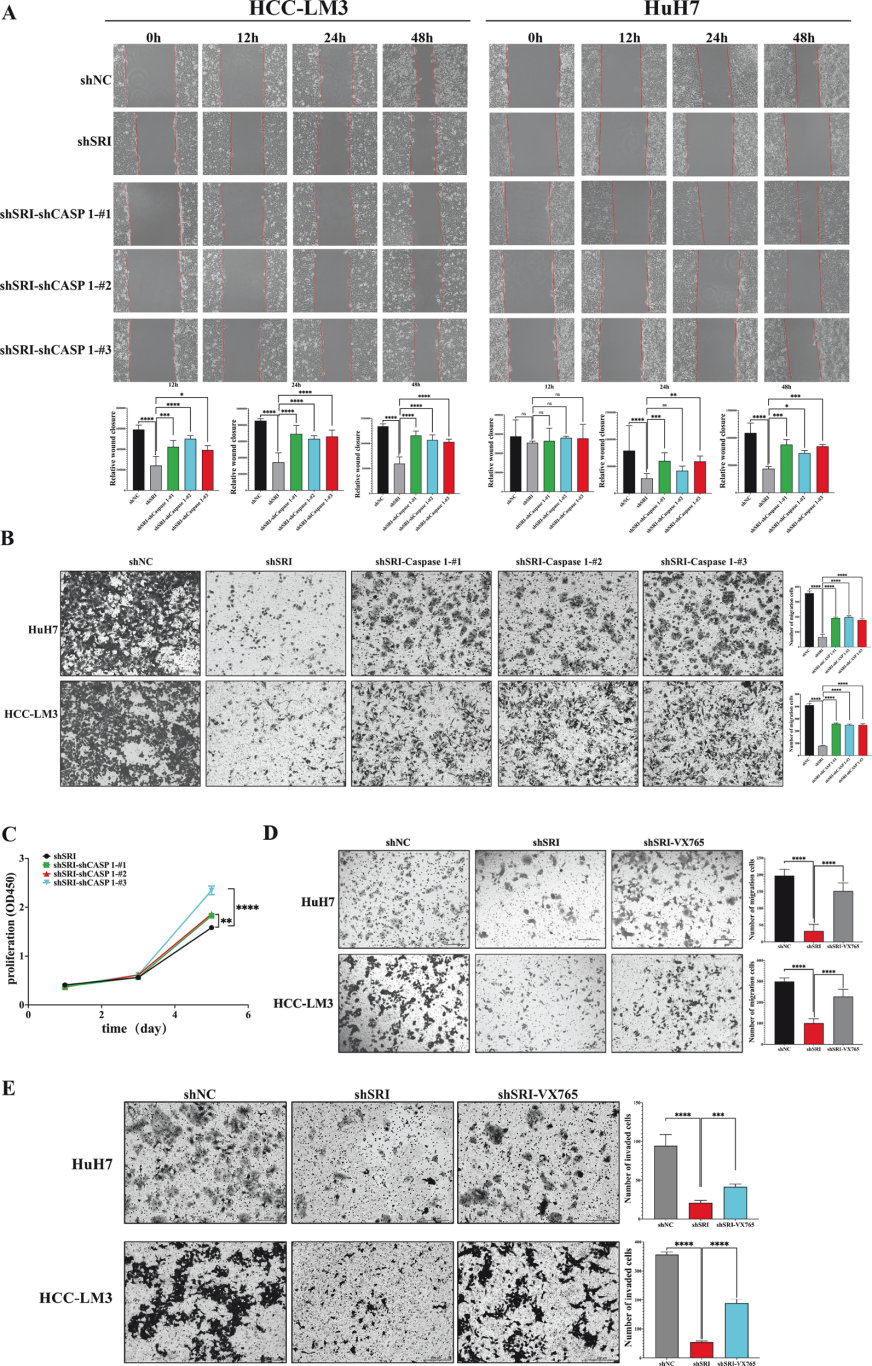
Inhibition of Caspase-1 reversed the inhibitory effect of Sorcin knockdown on the malignant progression of HCC. **A** Representative images of wound-healing assays of HCC-LM3 and HuH-7 cells showed that Caspase-1 knockdown promoted the migration of Sorcin inhibition-induced suppression cell migration at 0 h,12 h, 24 h, and 48 h. **B** Transwell invasion assays showed that suppression of Cspase-1 promoted cell invasion in HCC-LM3 and Huh-7 cells. **C** The CCK8 cell proliferation assays demonstrated Caspase-1 knockdown promoted the proliferation ability of HCC cells. **D** HCC-LM3 and HuH7 cells of Sorcin knockdown with shRNA interference were pre-treated with 10 μg/mL VX765 for 24 h, and then the transwell assays revealed that inhibition of Caspase-1 expression by VX765 promoted cell migration. **E** The transwell invasion assays revealed that Sorcin knockdown inhibited the invasion ability of HCC cells, and VX765 treatment partially restored the invasion ability. One-way ANOVA was used for multiple comparisons; ^*^P < 0.05, ^**^P < 0.01, ^***^P < 0.001, ^****^P < 0.0001, and ns no significance.

Wound-healing and transwell assays manifested that the migration ability of shSRI-shCASP1 cells was higher than that of the corresponding Sorcin-silenced group (Fig. 7A, B). Furthermore, the CCK-8 assays demonstrated that the proliferation ability of HCC cells in the shSRI-shCASP1 group was higher than that of the Sorcin knockdown group (Fig. 7C). Similarly, transwell assays also revealed that VX765 partially reversed the inhibitory effect of Sorcin knockdown on the migration and invasion ability of HCC cells (Fig. 7D, E).

### Sorcin promotes tumor growth by inhibiting the activation of Caspase1-dependent pyroptosis in vivo

**Fig. 8:**
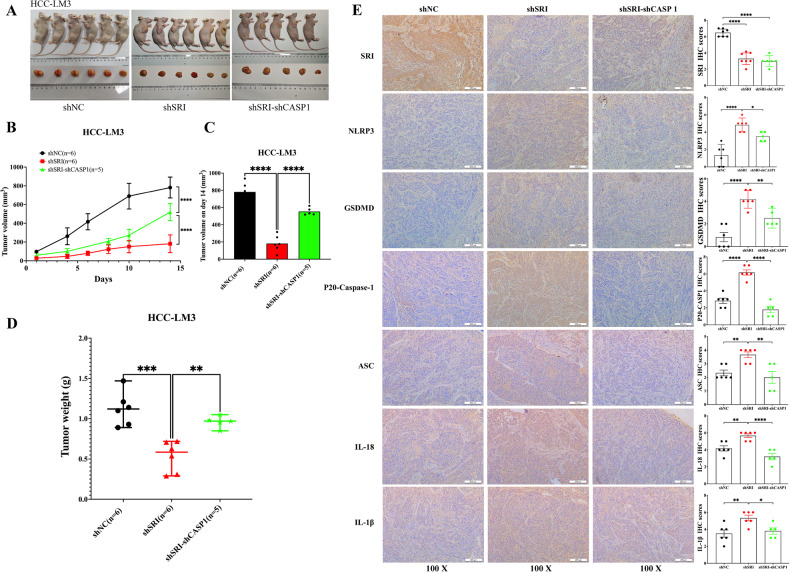
The effect of Sorcin inhibition-induced activation of Caspase-1 on tumor growth in vivo. **A** The representative morphology of tumors in nude mice. **B** The nude mice were injected subcutaneously with HCC-LM3 cells of Sorcin knockdown into the armpit of nude mice in each group followed by monitoring growth for three weeks. **C** Differences in tumor volumes on day 14 among the three groups. **D** The differences in tumor weight in various groups when mice were sacrificed. **E** Immunohistochemical analysis of tumor tissues in each group verified the expression of pyroptosis-related proteins, including NLRP3, GSDMD, P20-Caspase-1, ASC, IL-18, and IL-1β. One-way ANOVA was used for multiple comparisons; ^*^P < 0.05, ^**^P < 0.01, ^***^P < 0.001, and ^****^P < 0.0001.

The subcutaneous tumor model of nude mice was constructed by injecting Sorcin into the armpit of nude mice. The data showed that the volume and weight of the tumors formed in the Sorcin knockdown group were significantly lower than those of the tumors in the group treated with the negative control, while compared to the shSRI group, the shSRI-shCASP1 group was able to partially restore its growth capacity (Fig. 8A–D). Furthermore, the immunohistochemistry results showed that compared with the control group, the shSRI group exhibited increased expression of pyroptosis-related proteins, whereas compared with the shSRI group, the expression of pyroptosis-related proteins was decreased in the shSRI-shCASP1 group (Fig. 8E). Consistent with the results obtained in vitro, Sorcin contributed to aggressive biological behaviors by inhibiting the activation of the Caspase-1-dependent pyroptosis pathway.

## Discussion

Despite recent advances in the treatment of HCC, the prognosis of HCC patients remains poor because most patients have advanced HCC when diagnosed, and advanced HCC has multidrug resistance to cytotoxic chemotherapy drugs, which brings many obstacles to the clinical diagnosis and treatment of advanced HCC [32, 33]. Hence, finding new therapeutic targets and exploring the molecular mechanism behind their occurrence is essential in the fight against liver cancer and predicting its prognosis.

Sorcin is a regulatory protein that can exert its oncogenic activity by regulating the expression of various molecules in biological systems and inducing different signaling pathways [12, 14, 34]. It appears that dysregulated Sorcin expression is closely linked to the occurrence, invasion, metastasis, and progression of cancer in numerous human tumors [7, 9–11, 35, 36]. In our study, Sorcin expression is highly correlated with poor prognosis and may increase recurrence and metastasis risks in HCC, thus, Sorcin plays a promotive role in HCC progression. Furthermore, we observed that Sorcin-silenced significantly inhibited the migration, invasion, and proliferation of HCC cells. Thus, Sorcin has potential prognostic value and provides new insights into the identification of novel biomarkers associated with HCC progression and prognosis.

Recent evidence suggests that pyroptosis in tumor cells is induced, leading to a strong inflammatory response and significant tumor regression [29, 37]. NLRP3 inflammasome is a key component of classical pyroptosis progression, promotes the maturation of pro-IL-18 and pro-IL-1β and triggers pyroptosis by activating Caspase-1 in response to stimulation by various pathogen-stimulating factors or lipopolysaccharides [23]. Activation of pyroptosis can induce effective antitumor activity. For example, NLRP3 inflammasome-induced pyroptosis significantly inhibited the growth characteristics and metastatic potential of HCC cells [38]. This is consistent with our findings that Sorcin-silenced promotes upregulation of the expression of pyroptosis-related proteins, thereby inhibiting the proliferation, migration, and invasion of HCC cells.

Apoptosis has been reported to be an important process in inhibiting metastasis [39], and ferroptosis is also a critical tumor suppressor mechanism [40]. Tumor cells can survive under hypoxic or stressful conditions through the process of autophagy, which can reduce tumor necrosis and contribute to subsequent immune cell infiltration and metastasis [41]. Thus, programmed cell death, including pyroptosis, apoptosis, ferroptosis, and autophagy, plays an increasingly important role in the progression, recurrence, and metastasis of tumors [42]. Our study revealed that Sorcin promotes HCC progression by regulating the pyroptosis pathway but has no significant effect on apoptosis, ferroptosis, and autophagy progression in HCC cells.

We identified NLRP3 inflammasome as Sorcin-interacting proteins. NLRP3 inflammasome has previously been shown to mediate the production of IL-18, which increases the tumoricidal activity of natural killer cells against metastatic colon tumor cells in mouse liver [43]. Wei Q et al. also proved that NLRP3 inflammasome components are significantly downregulated and involved in HCC progression [44], which is consistent with our findings. Thus, NLRP3 plays a protective role in HCC progression. In our study, Sorcin negatively regulates NLRP3 inflammasome in HCC cells, likely because of the interaction of highly expressed Sorcin with NLRP3 inflammasome, interfering with the activation and assembly of NLRP3 inflammasome, which are essential for host defense against pathogen invasion and maintenance of homeostasis, and thus decreasing host defense against pathogen invasion, resulting in weakened immune protection of the host.

Additionally, NLRP3-upregulated 17β-estradiol (E2) significantly inhibited the malignant biological behavior of HCC cells through the E2/ERβ/MAPK pathway, further demonstrating the protective effect of NLRP3 in HCC [45]. Lee G-S et al. showed that NLRP3 inflammasomes are activated by Ca^2+^ via calcium-sensing receptors; thus, Ca^2+^ serves as a key molecular regulator of inflammasomes [46]. Sorcin is a phosphorylated calcium-binding protein that plays an integral role in the regulation of calcium homeostasis and calcium balance [47–49]. Therefore, high expression of Sorcin in HCC cells might have a competitive binding Ca^2+^ with NLRP3 inflammasome, weakening the promotion effect of Ca^2+^ on NLRP3 inflammasome activation, inhibiting the activation and assembly of NLRP3 inflammasome, further preventing the activation of the natural immune response in vivo, and suppressing the immune protective effect of the body. Thus, Sorcin exerts its oncogenic effect by interacting with NLRP3 inflammasome and inhibits the protective effect of NLRP3 inflammasome on HCC.

It was previously shown that activation of pyroptosis inhibited HCC proliferation and invasive metastasis, and this effect was attenuated by caspase-1 inhibitors [38], which was consistent with our findings. Our results manifested that the inhibitory effects of Sorcin knockdown on HCC cell proliferation, migration, and invasion were reversed by using the Caspase-1 inhibitor VX765 and the NLRP3 inhibitor MCC950, further suggesting that Sorcin facilitates HCC progression by regulating NLRP3 inflammasome-mediated classical pyroptosis. Furthermore, NLRP3 inflammasome is mainly expressed in immune cells, and high Sorcin expression might inhibit the tumoricidal activity of immune cells against tumor cells and the protective effect of NLRP3 inflammasome against HCC. Moreover, Sorcin-silenced activates pyroptosis and may change the immunosuppressive environment in which the tumor tissue is located to an immune-activating environment, where immune cells will be continuously recruited to the tumor site. Thus, the combination of suppression of Sorcin expression with immune checkpoint blockade therapy in clinical HCC treatment has the potential to achieve a more effective tumor-killing effect. Combining chemotherapy and immunotherapy enhances tumor suppression and provides new insights into clinical HCC treatment.

In summary, Sorcin is highly expressed in human HCC tissues and cell lines and negatively regulates the expression of NLRP3 inflammasome by interacting with NLRP3 inflammasome to inhibit the activation of pyroptosis and promoting the proliferation, migration, and invasion of HCC. This study provides a novel biomarker and a new therapeutic approach for predicting and treating HCC, which has potential clinical application and requires further clinical research to validate and develop.

### Supplementary information

Reproducibility checklist

Supplementary Figure legends

Supplemental Table

Supplementary Figure S1

Supplementary Figure S2

Supplementary Figure S3

Supplementary Figure S4

Supplementary Figure S5

original western blots

## Data Availability

All data generated or analyzed during this study are included in this published article and its supplementary information files. Further inquiries can be directed to the corresponding author.
