# Correction: Sorcin regulate pyroptosis by interacting with NLRP3 inflammasomes to facilitate the progression of hepatocellular carcinoma

**DOI:** 10.1038/s41419-024-06604-x

**Published:** 2024-07-02

**Authors:** Zhenfen Li, Ziyue Yang, Yuanyuan Zhu, Chunmeng Fu, Ning Li, Fang Peng

**Affiliations:** 1 Department of Blood Transfusion, Clinical Transfusion Research Center, Xiangya Hospital, Central South University, Changsha, Hunan China; 2 National Health Commission (NHC) Key Laboratory of Cancer Proteomics, Xiangya Hospital, Central South University, Changsha, Hunan China; 3 National Clinical Research Center for Geriatric Disorders, Xiangya Hospital, Central South University, Changsha, China

**Keywords:** Liver cancer, Metastasis, Tumour biomarkers

Correction to: *Cell Death and Disease* (2023) 14:678 10.1038/s41419-023-06096-1, published online 13 October 2023

We reported that inhibition of NLRP3 by MCC950 and Caspase1 by VX765 in the HCCLM3-shSRI restores tumor migration function. We conducted two groups of experiments simultaneously. Group 1 was HCCLM3-shNC, shSRI, shSRI-MCC950 (showed in Fig. 5E), and Group 2 was HCCLM3-shNC, shSRI, shSRI-VX765 (showed in Fig. 7D). The Negative controls for the entire two groups of the experiment were HCCLM3-shNC, which were the same cells without any gene interference and reagent stimulation. We mistakenly used the Group1 HCCLM3-NC image in Fig. 7D in the published version. Although the HCCLM3-NC image in Fig. 7D needs to be changed, the descriptions in figure legends and the results of Fig. 7D are still correct.
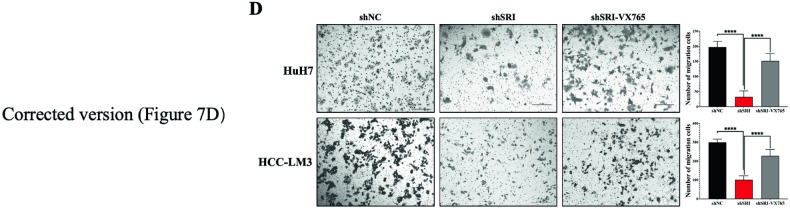


The original article has been corrected.

