# Supplementary material for: Sorcin regulate pyroptosis by interacting with NLRP3 inflammasomes to facilitate the progression of hepatocellular carcinoma
Source: Cell Death Dis. 2023 Oct 13;14(10):678. doi: 10.1038/s41419-023-06096-1 (PMC10575890; doi:10.1038/s41419-023-06096-1)
Supplement: Supplementary file 2 — Supplementary Figure legends [file 41419_2023_6096_MOESM2_ESM.docx]

**Supplementary Figure legends**

**Figure S1: Sorcin expression in tumor tissues and paired normal tissues in various cancers according to the RNA sequencing data from the TCGA database via the SangerBox online tool.** The expression of Sorcin in liver hepatocellular carcinoma (LIHC) is higher (n = 369) than in normal liver tissues (n = 160) (****: *P* < 0.0001).

**Figure S2: Overexpressing and silencing Sorcin in hepatoma cell lines.**

(A-B) The downregulation of Sorcin in HuH7 and HCC-LM3 cells after lentivirus-mediated silence of SRI was confirmed by western blot and the statistics were shown in histograms. (E-F) Sorcin knockdown in HCC-LM3 and HuH7 cells was verified by qRT-PCR. (C, D, G, H) Stably transfected HCC-LM3 and HuH7 cell lines overexpressed Sorcin was successfully constructed and verified by Western blot and qRT-PCR. **: *P* < 0.01, ***: *P* < 0.001, and ****: *P* < 0.0001.

**Figure S3: Enrichment of functions and signaling pathways analysis were performed based on Gene Ontology (GO) and the Kyoto Encyclopedia of Genes and Genomes (KEGG) database.**

**Figure S4: Bioinformatics analysis showed that Caspase-1 (r^2^= -0.29, *P* < 0.01) and NLRP3 (r^2^= -0.35, *P* < 0.01) expression levels were negatively correlated with Sorcin expression in HCC tissues.**

**Figure S5: Sorcin negatively regulated the expression of NLRP3 and Caspase-1 in HCC cell lines with Sorcin knockdown and overexpression.**

**Table S1: Oligonucleotides used in knockdown shRNA studies.**

**Table S2: Oligonucleotides used in real-time PCR.**

**Table S3: A description of the antibodies used in this study.**

**Table S4：Relationships between Sorcin expression and clinicopathological factors in thirty HCC samples and adjacent nontumorous tissue from HCC patients.**
