# Supplementary material for: Sorcin regulate pyroptosis by interacting with NLRP3 inflammasomes to facilitate the progression of hepatocellular carcinoma
Source: Cell Death Dis. 2023 Oct 13;14(10):678. doi: 10.1038/s41419-023-06096-1 (PMC10575890; doi:10.1038/s41419-023-06096-1)
Supplement: Supplementary file 3 — Supplemental Table [file 41419_2023_6096_MOESM3_ESM.docx]

**Table S1: Oligonucleotides used in knockdown shRNA studies**

| Primer Name | sequences (5' to 3') |
| --- | --- |
| shSRI-#1 | GATCCGCGGACAAACTCAGGATCCGCTGTATCTCGAGATACAGCGGATCCTGAGTTTGTCCGTTTTTTG |
| shSRI-#2 | GATCCGCCCAGGCTGTGAATTCAATTGCAAACTCGAGTTTGCAATTGAATTCACAGCCTGGGTTTTTTG |
| shSRI-#3 | GATCCGCAGCTTTCGAAGACGGGATACTGCTCTCGAGAGCAGTATCCCGTCTTCGAAAGCTGTTTTTT |
| shCASP1-#1 | GGCTTACTGGATGAATTATTATTCAAGAGATAATAATTCATCCAGTAAGCCTTTTTT |
| shCASP1-#2 | GGCAGAGATTTATCCAATAATTTCAAGAGAATTATTGGATAAATCTCTGCCTTTTTT |
| shCASP1-#3 | GTTTGAGGATGATGCTATTAATTCAAGAGATTAATAGCATCATCCTCAAACTTTTTT |

**Table S2: Oligonucleotides used in real-time PCR**

| Gene | Primer sequences (5' to 3') | |
| --- | --- | --- |
|  | Forward | Reverse |
| CASP3 | TCGGTCTGGTACAGATGTCG | CATCACGCATCAATTCCACAATTTC |
| CASP1 | TAATGGACAAGTCAAGCCGCA | ACCCCAGATTTTGTAGCAGCA |
| GSDME | TTCGTGCTCTGTCTGATGATGG | GCAAACAAGCGCTGCACAAT |
| GSDMD | GGTAGTCCGGAGAGTGGTCC | CTTCCTAACCACCAGGCAGT |
| NLRP3 | AGGAGGACTTCGTGCAAAGG | CAGTGACTCCACCCGATGAC |
| SRI | CTGGGCTGTACTGAATGGCTG | CATTGTTGTCAGGGCCTTCTG |
| IL-18 | GAGAAGTGTCCCAGGACATGATA | ATCCCCCAATTCATCCTCTTTT |
| IL-1β | GCTACGAATCTCCGACCACC | TGTCCATGGCCACAACAACT |
| GPX4 | CCAGTGAGGCAAGACCGAAG | TTCCCGAACTGGTTACACGG |
| SLC7A11 | GAAATGTTAACGGGAGGCTGC | GCTCCAATGATGGTGCCAATG |
| Beclin 1 | AACCAGATGCGTTATGCCCA | TGTGGAAGGTTGCATTAAAGACG |

**Table S3: A description of the antibodies used in this study**

| Number | Antibody target genes | Catalog | Dilution | Company |
| --- | --- | --- | --- | --- |
| 1 | SRI | 16471-1-AP | 1:1000 | Proteintech |
| 2 | NLRP3 | ab263899 | 1:1000 | ABCAM |
| 3 | NLRP3 | 19771-1-AP | 1:1000 | Proteintech |
| 4 | ASC/TMS1 | 10500-1-AP | 1:1000 | Proteintech |
| 5 | Caspase-1/p20/p10 | 22915-1-AP | 1:1000 | Proteintech |
| 6 | P20-Caspase-1 | YC0022 | 1:1000 | Immunoway |
| 7 | pro-IL18 | 10663-1-AP | 1:5000 | Proteintech |
| 8 | IL-18 | YN1926 | 1:1000 | Immunoway |
| 9 | pro-IL-1β | 16806-1-AP | 1:1000 | Proteintech |
| 10 | IL-1β | YT5201 | 1:1000 | Immunoway |
| 11 | GSDMD | 20770-1-AP | 1:1000 | Proteintech |
| 12 | GSDMD-N | YT7991 | 1:1000 | Immunoway |
| 13 | GSDME | R24107 | 1:1000 | ZEN-BIOSCIENCE |
| 14 | Caspase 3 | 19677-1-AP | 1:1000 | Proteintech |
| 15 | BAX | 50599-2-Ig | 1:1000 | Proteintech |
| 16 | SLC7A11 | 26864-1-AP | 1:1000 | Proteintech |
| 17 | GPX4 | 67763-1-Ig | 1:1000 | Proteintech |
| 18 | Flag | M20008 | 1:5000 | Abmart |
| 19 | GAPDH | M20006 | 1:5000 | Abmart |

**Table S4：Relationships between Sorcin expression and clinicopathological factors in thirty HCC samples and adjacent nontumorous tissue from HCC patients**

|  | **Tissues** | **N** | **IHC scores** | | | | | |
| --- | --- | --- | --- | --- | --- | --- | --- | --- |
|  |  |  | <3.9 | 4~4.9 | 5~5.9 | 6.0~7.0 | P-value | |
| **Gender** |  |  |  |  |  |  |  |  |
|  | male | 27 | 3 | 2 | 5 | 17 |  | 0.1949 |
|  | female | 3 | 0 | 0 | 0 | 3 |  |  |
| **Age** |  |  |  |  |  |  |  |  |
|  | ≤60 | 16 | 1 | 0 | 3 | 12 |  | 0.151 |
|  | >60 | 14 | 2 | 2 | 2 | 8 |  |  |
| **Differentiation** |  |  |  |  |  |  |  |  |
|  | poorly | 5 | 0 | 0 | 3 | 2 |  |  |
|  | moderately | 16 | 1 | 2 | 2 | 11 | poorly vs. moderately | *0.0071 |
|  | highly | 9 | 2 | 0 | 0 | 7 | poorly vs. highly | *0.0016 |
| **Tumor size** |  |  |  |  |  |  |  |  |
|  | ≤5cm | 16 | 1 | 1 | 3 | 11 |  | 0.4542 |
|  | >5cm | 14 | 2 | 1 | 2 | 9 |  |  |
| **Microvascular Invasion** |  |  |  |  |  |  |  |  |
|  | M0 | 17 | 1 | 0 | 3 | 13 |  |  |
|  | M1 | 12 | 2 | 2 | 2 | 6 | M0 vs. M1 | 0.304 |
|  | M2 | 1 | 0 | 0 | 0 | 1 | M0 vs. M2 | 0.8222 |
| **Tumor** |  |  |  |  |  |  |  |  |
|  | T1b | 14 | 1 | 0 | 3 | 10 |  |  |
|  | T2 | 11 | 2 | 2 | 1 | 6 | T Ib vs. T 2 | 0.6059 |
|  | T3 | 1 | 0 | 0 | 0 | 1 | T Ib vs. T 3 | 0.8706 |
|  | T4 | 4 | 0 | 0 | 1 | 3 | T Ib vs. T 4 | 0.8739 |

Notes: P-value determined by Unpaired t-test, *: *p* < 0.05
