# Supplementary figures and images for: Sorcin regulate pyroptosis by interacting with NLRP3 inflammasomes to facilitate the progression of hepatocellular carcinoma
Source: Cell Death Dis. 2023 Oct 13;14(10):678. doi: 10.1038/s41419-023-06096-1 (PMC10575890; doi:10.1038/s41419-023-06096-1)

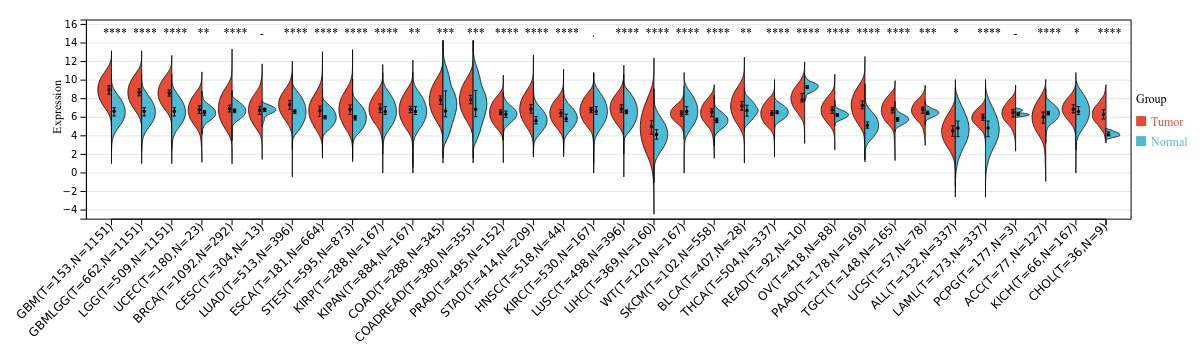

Supplement: Supplementary file 4 — Supplementary Figure S1 [file 41419_2023_6096_MOESM4_ESM.jpg]

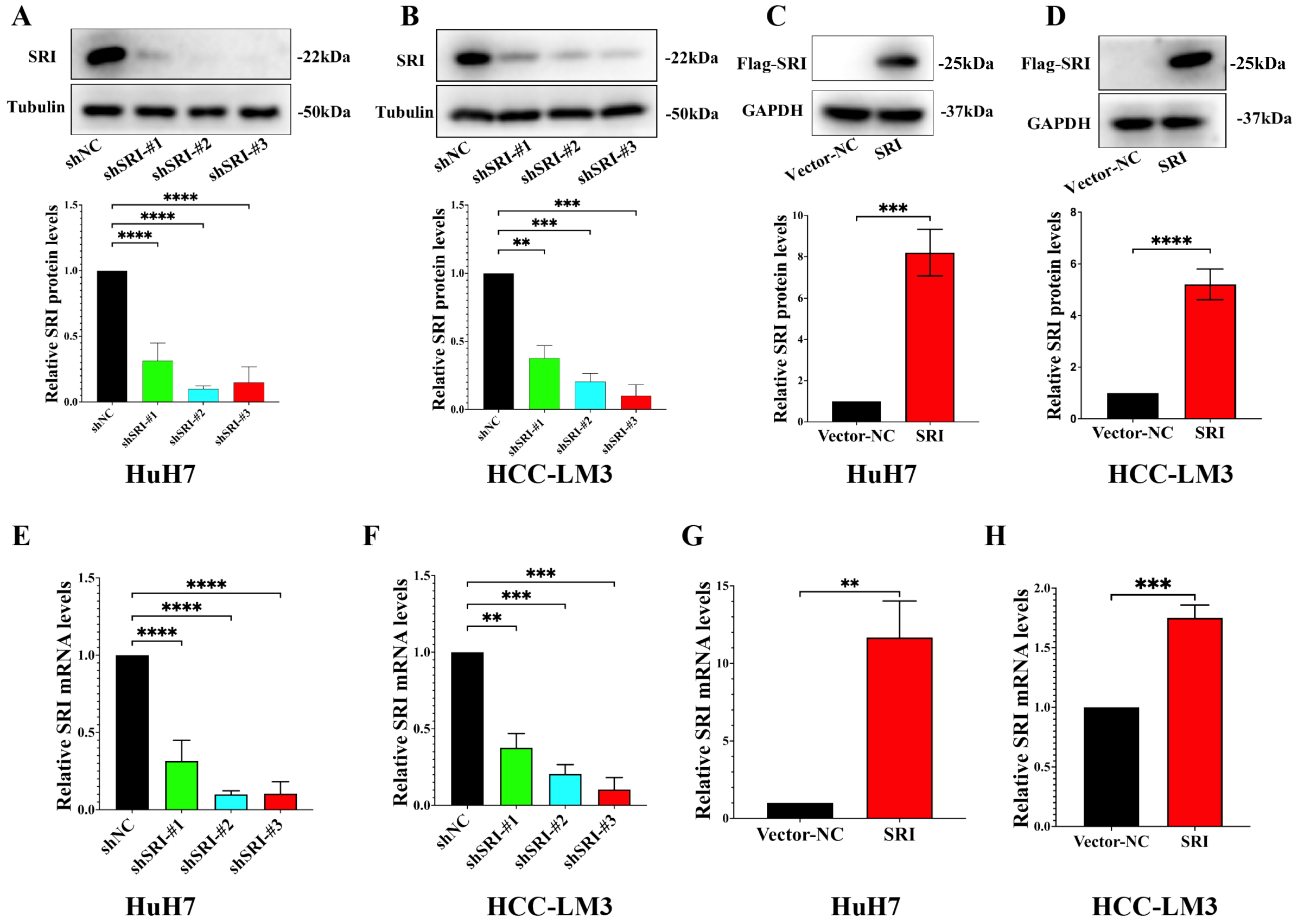

Supplement: Supplementary file 5 — Supplementary Figure S2 [file 41419_2023_6096_MOESM5_ESM.tif]

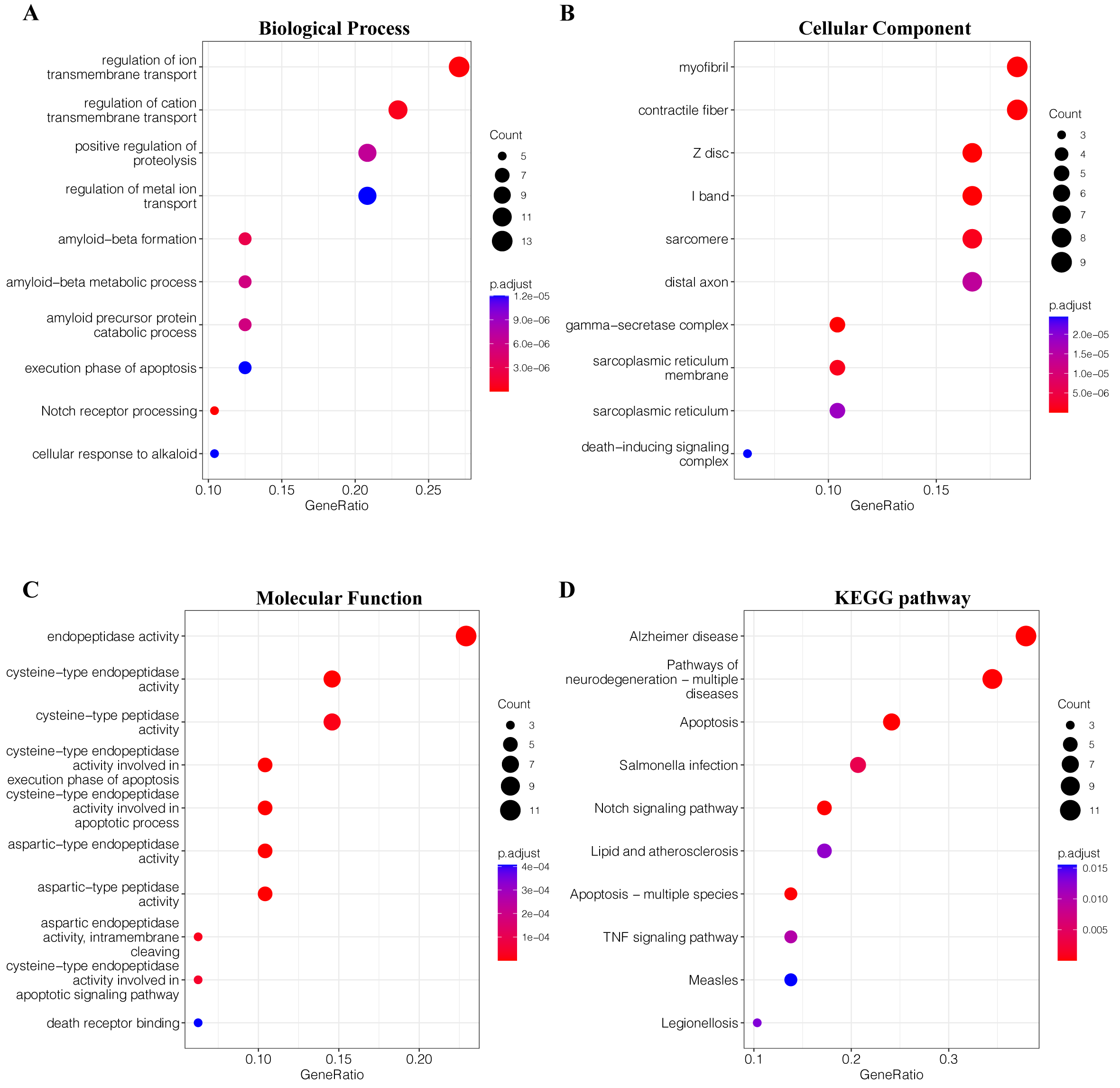

Supplement: Supplementary file 6 — Supplementary Figure S3 [file 41419_2023_6096_MOESM6_ESM.tif]

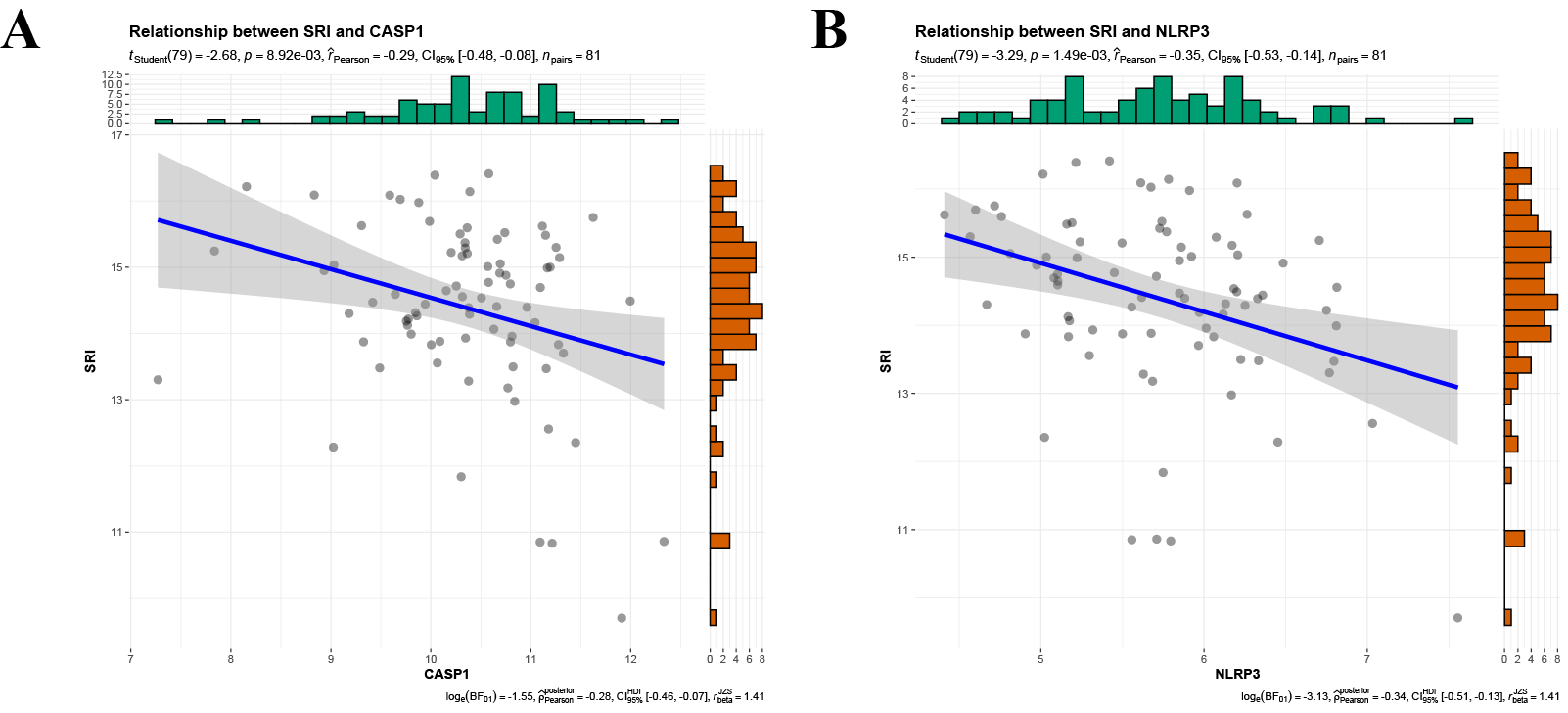

Supplement: Supplementary file 7 — Supplementary Figure S4 [file 41419_2023_6096_MOESM7_ESM.png]

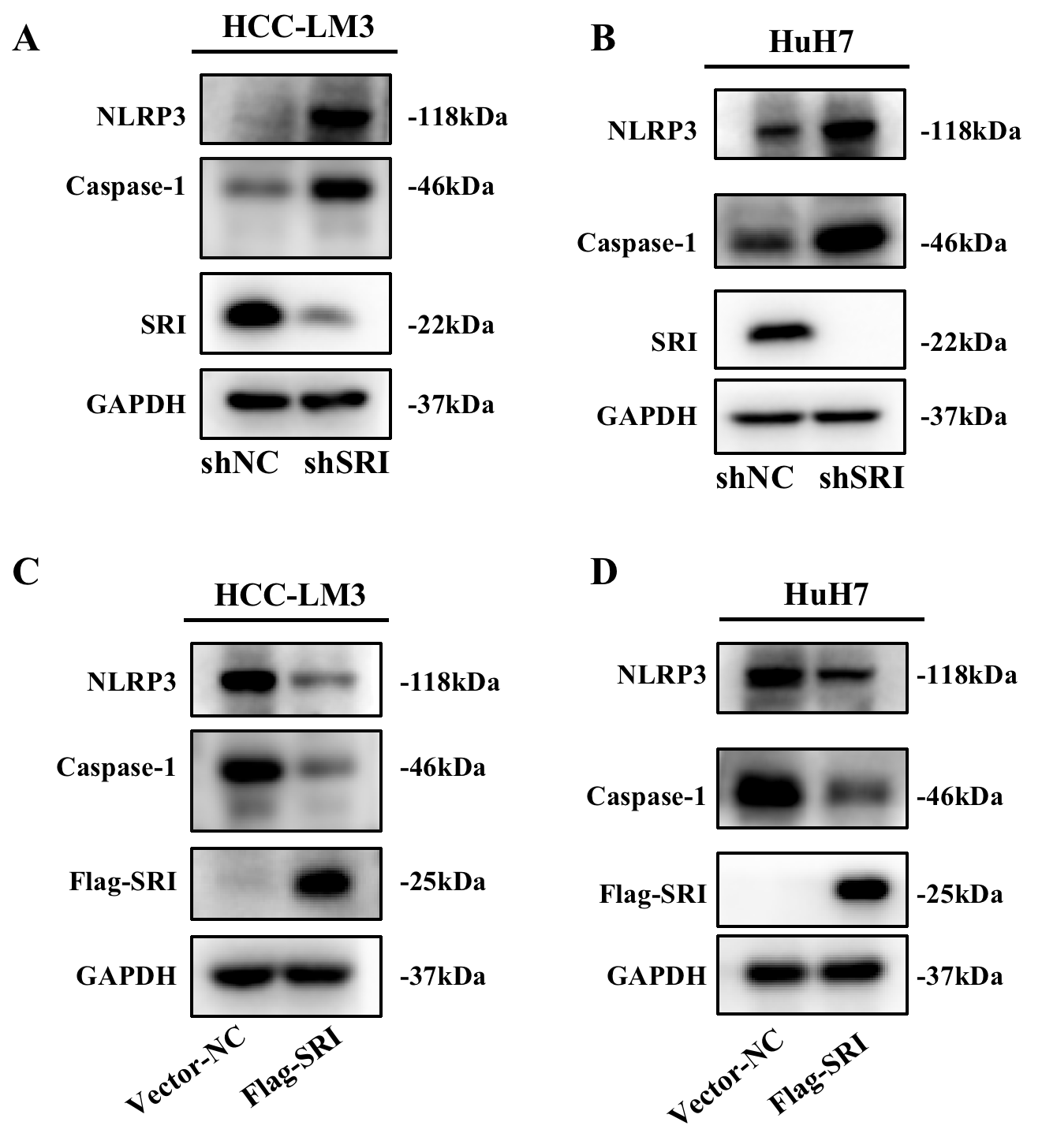

Supplement: Supplementary file 8 — Supplementary Figure S5 [file 41419_2023_6096_MOESM8_ESM.tif]
