# Supplementary material for: Sorcin regulate pyroptosis by interacting with NLRP3 inflammasomes to facilitate the progression of hepatocellular carcinoma
Source: Cell Death Dis. 2023 Oct 13;14(10):678. doi: 10.1038/s41419-023-06096-1 (PMC10575890; doi:10.1038/s41419-023-06096-1)

Fig.3 A HCC-LM3

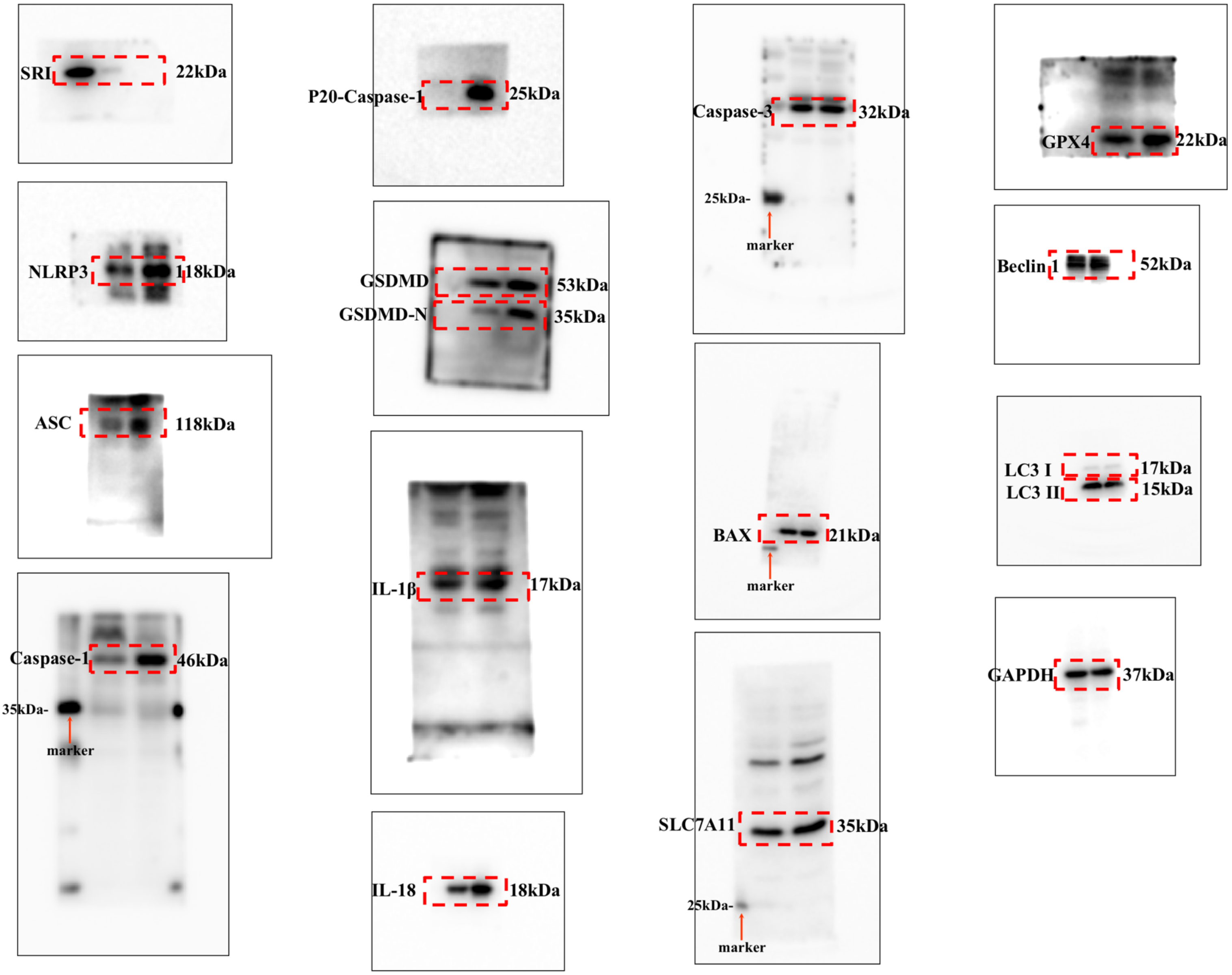

Fig.3 A HuH7

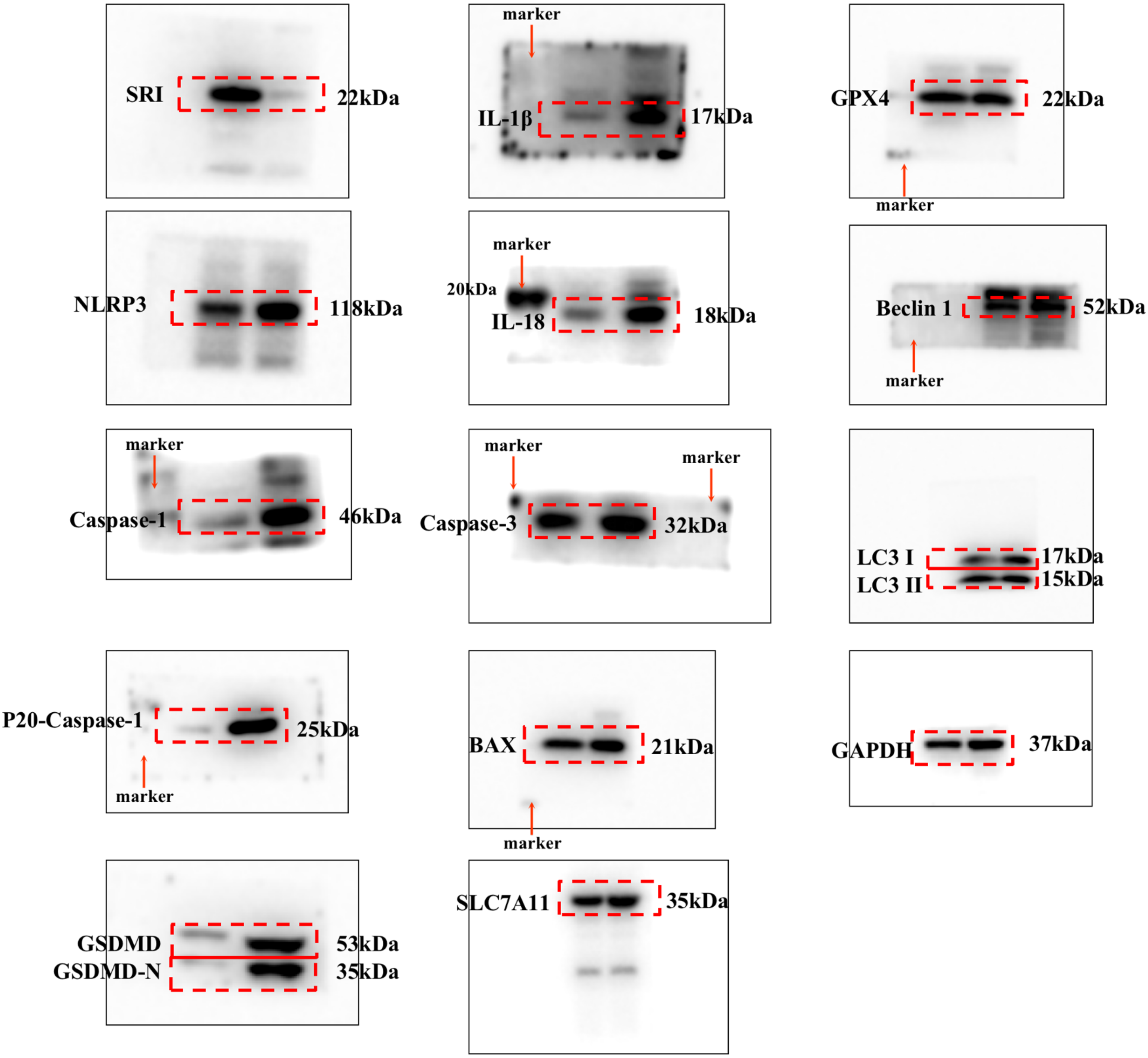

SRI
22kDa
marker
IL-1β
17kDa
GPX4
22kDa
marker
NLRP3
118kDa
marker
20kDa
IL-18
18kDa
Beclin 1
52kDa
marker
marker
Caspase-1
46kDa
marker
Caspase-3
marker
32kDa
LC3 I
17kDa
LC3 II
15kDa
P20-Caspase-1
25kDa
marker
BAX
21kDa
marker
GAPDH
37kDa
GSDMD
53kDa
GSDMD-N
35kDa
SLC7A11
35kDa

Fig.3 F HCC-LM3

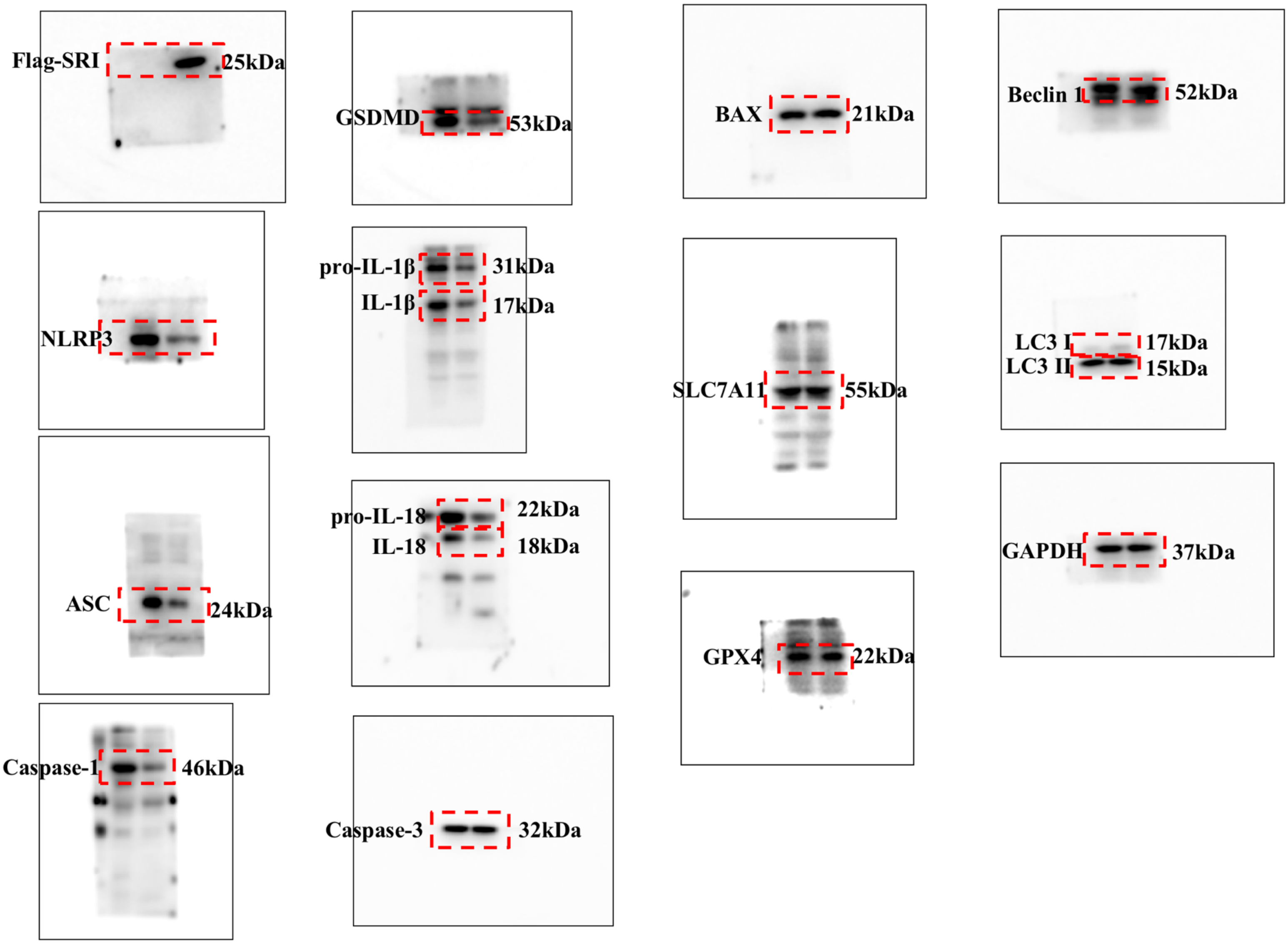

Fig.3 F HuH7

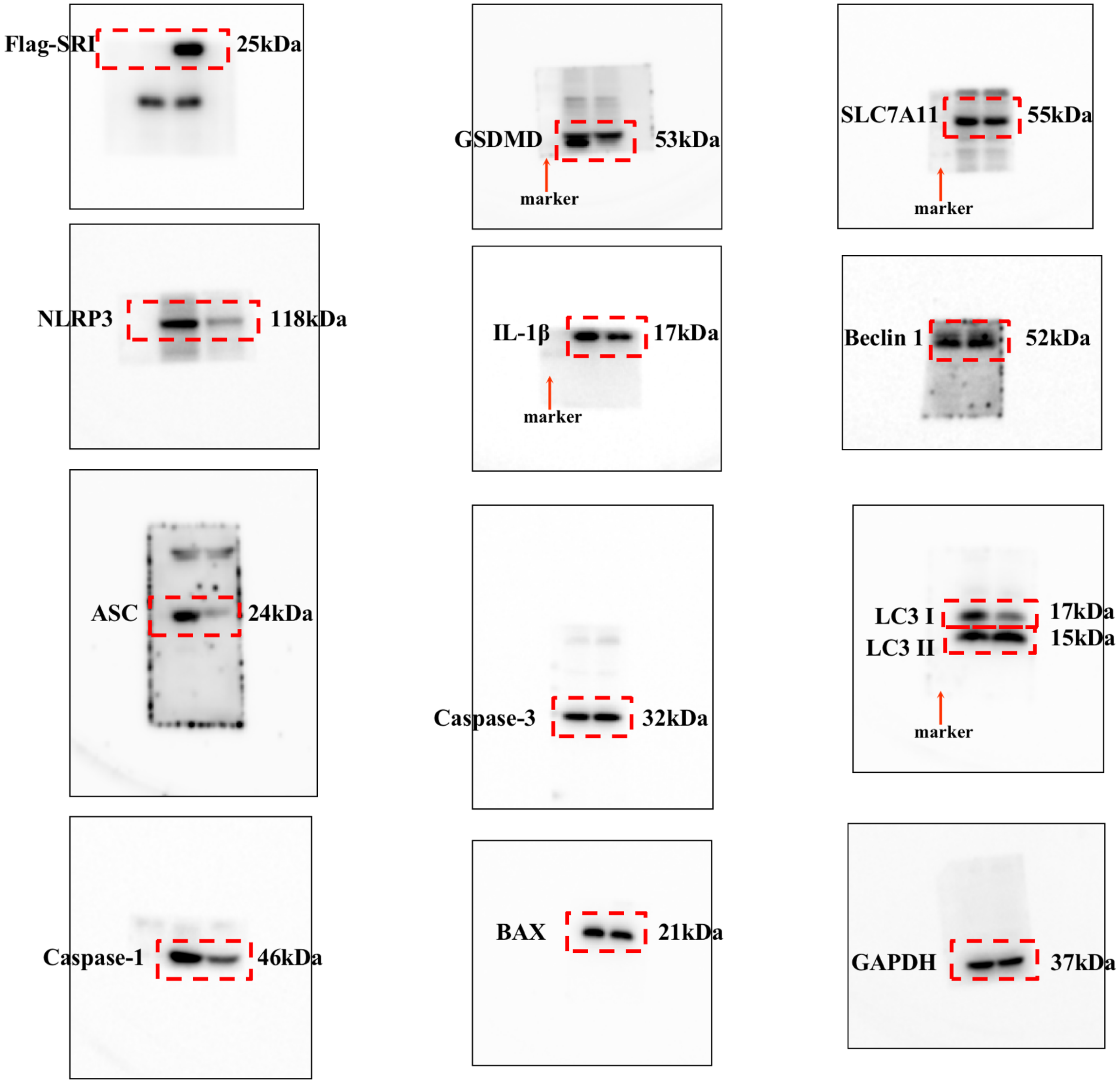
Flag-SRI
25kDa
GSDMD
53kDa
marker
SLC7A11
55kDa
marker
NLRP3
118kDa
IL-1β
17kDa
marker
Beclin 1
52kDa
ASC
24kDa
Caspase-3
32kDa
LC3 I
17kDa
LC3 II
15kDa
marker
Caspase-1
46kDa
BAX
21kDa
GAPDH
37kDa

Fig.4 B

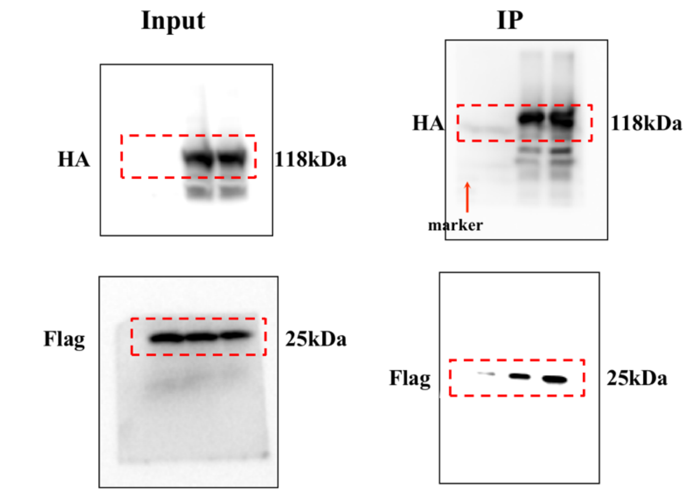

Fig.4 C

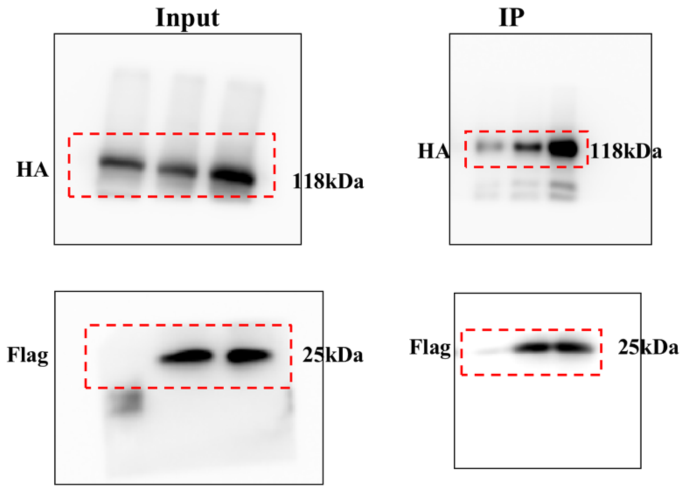

Fig.4 D

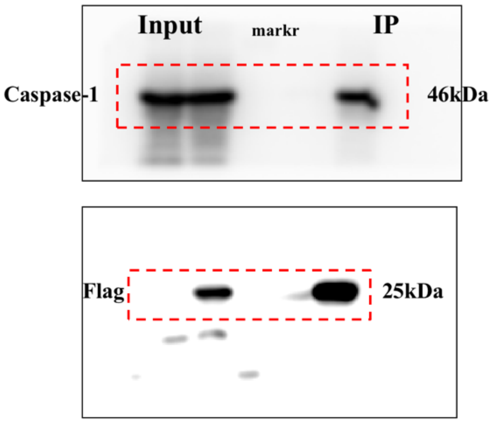

Fig.4 E

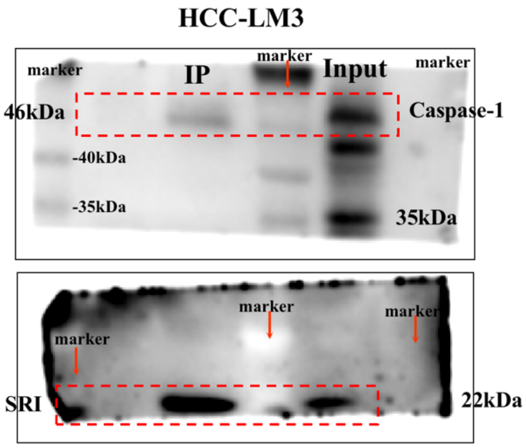

Fig.4 F

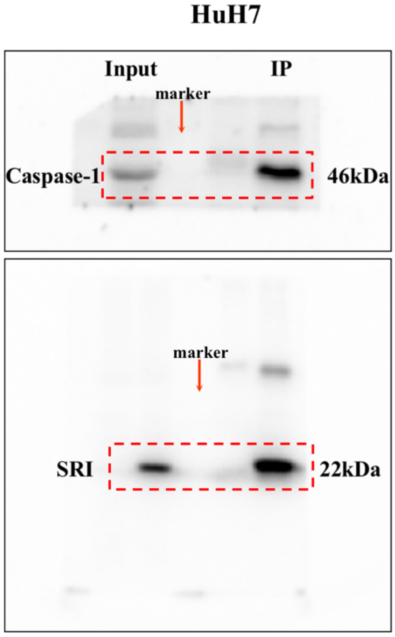

Fig.5 A HCC-LM3

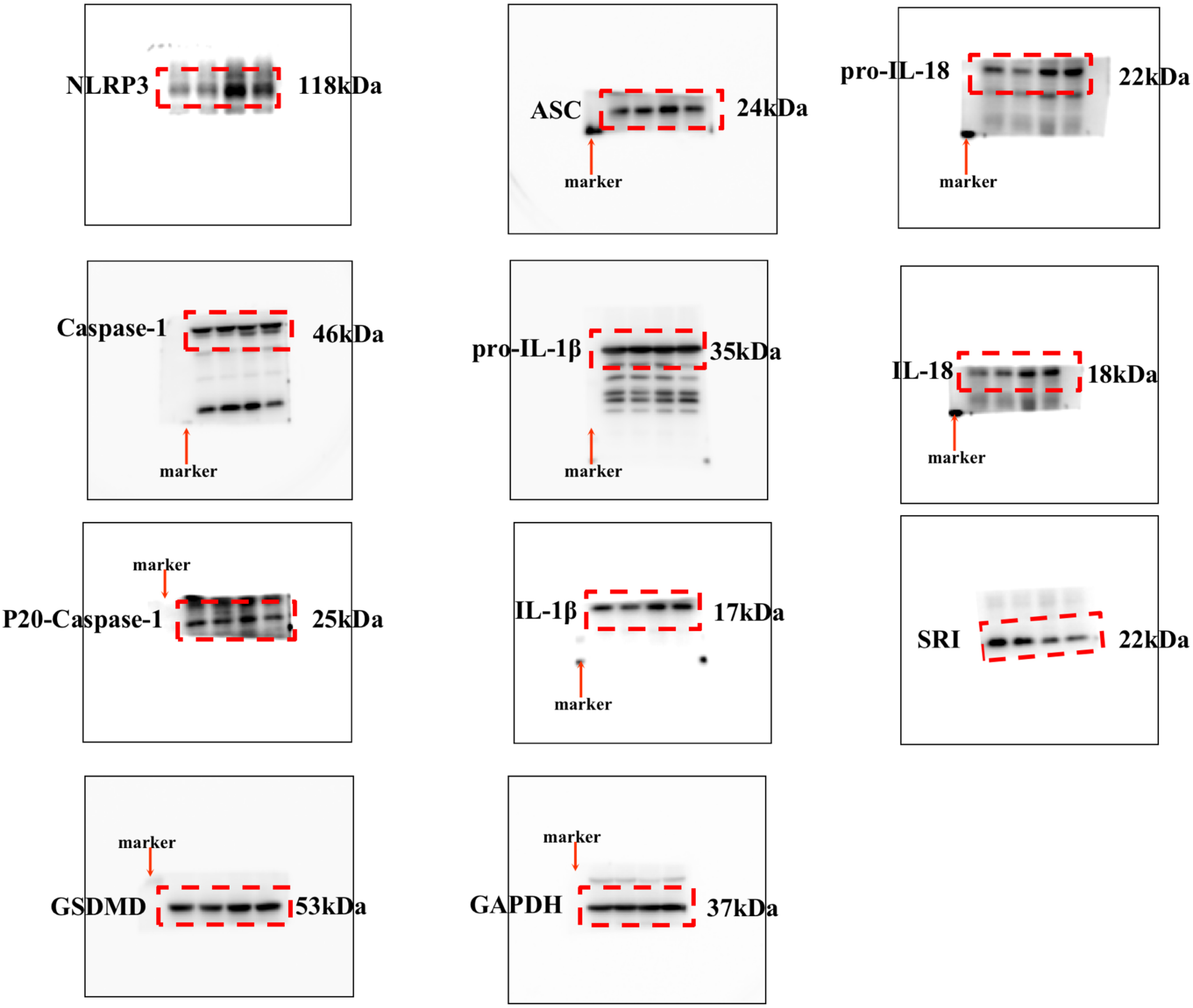

Fig.5 B HuH7

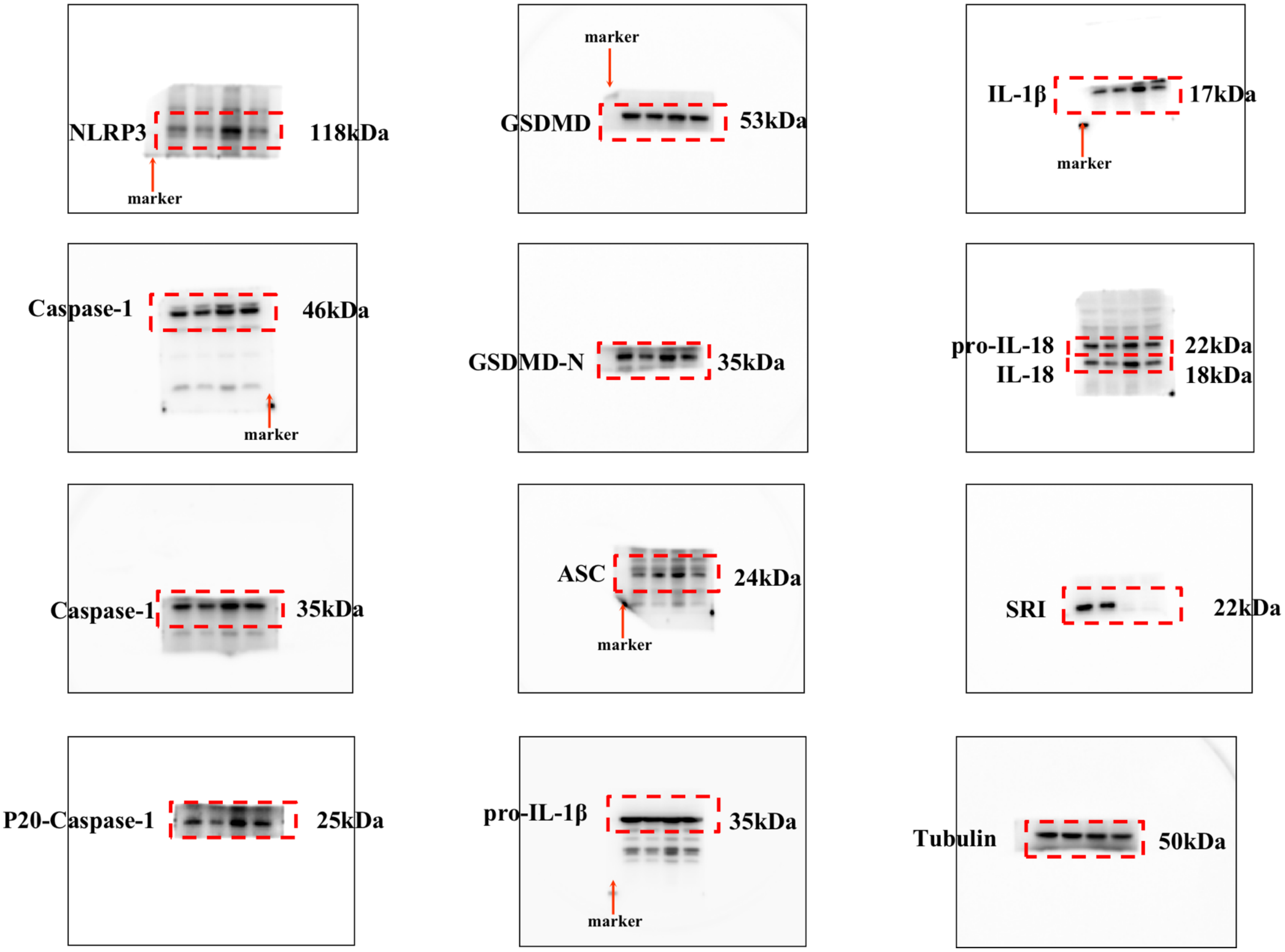
NLRP3
118kDa
marker
marker
GSDMD
53kDa
IL-1β
17kDa
marker
Caspase-1
46kDa
marker
GSDMD-N
35kDa
pro-IL-18
IL-18
22kDa
18kDa
Caspase-1
35kDa
ASC
24kDa
marker
SRI
22kDa
P20-Caspase-1
25kDa
pro-IL-1β
35kDa
marker
Tubulin
50kDa

Fig.6 A

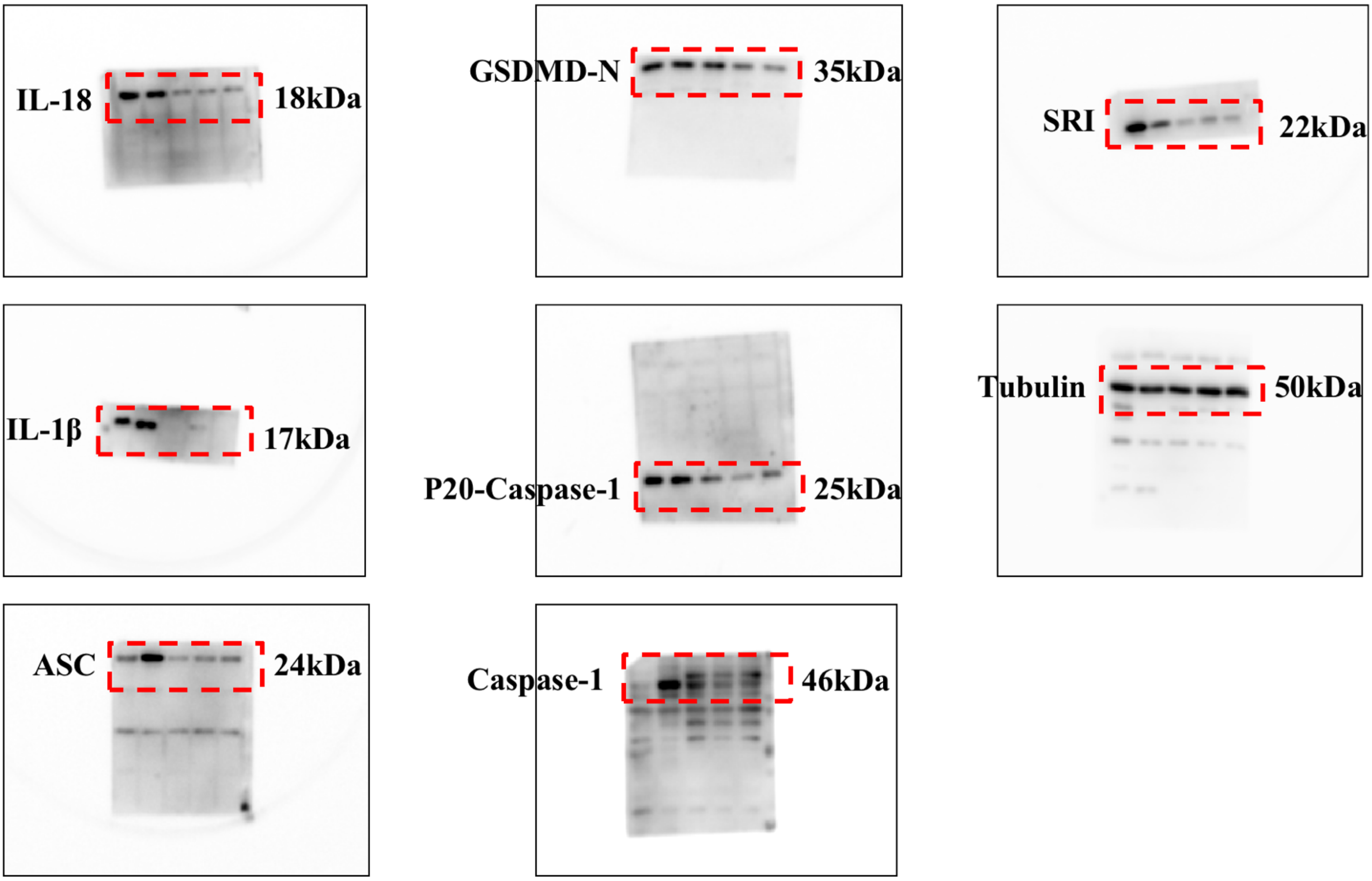

IL-18
18kDa
GSDMD-N
35kDa
SRI
22kDa
IL-1β
17kDa
P20-Caspase-1
25kDa
Tubulin
50kDa
ASC
24kDa
Caspase-1
46kDa

Fig.6 B

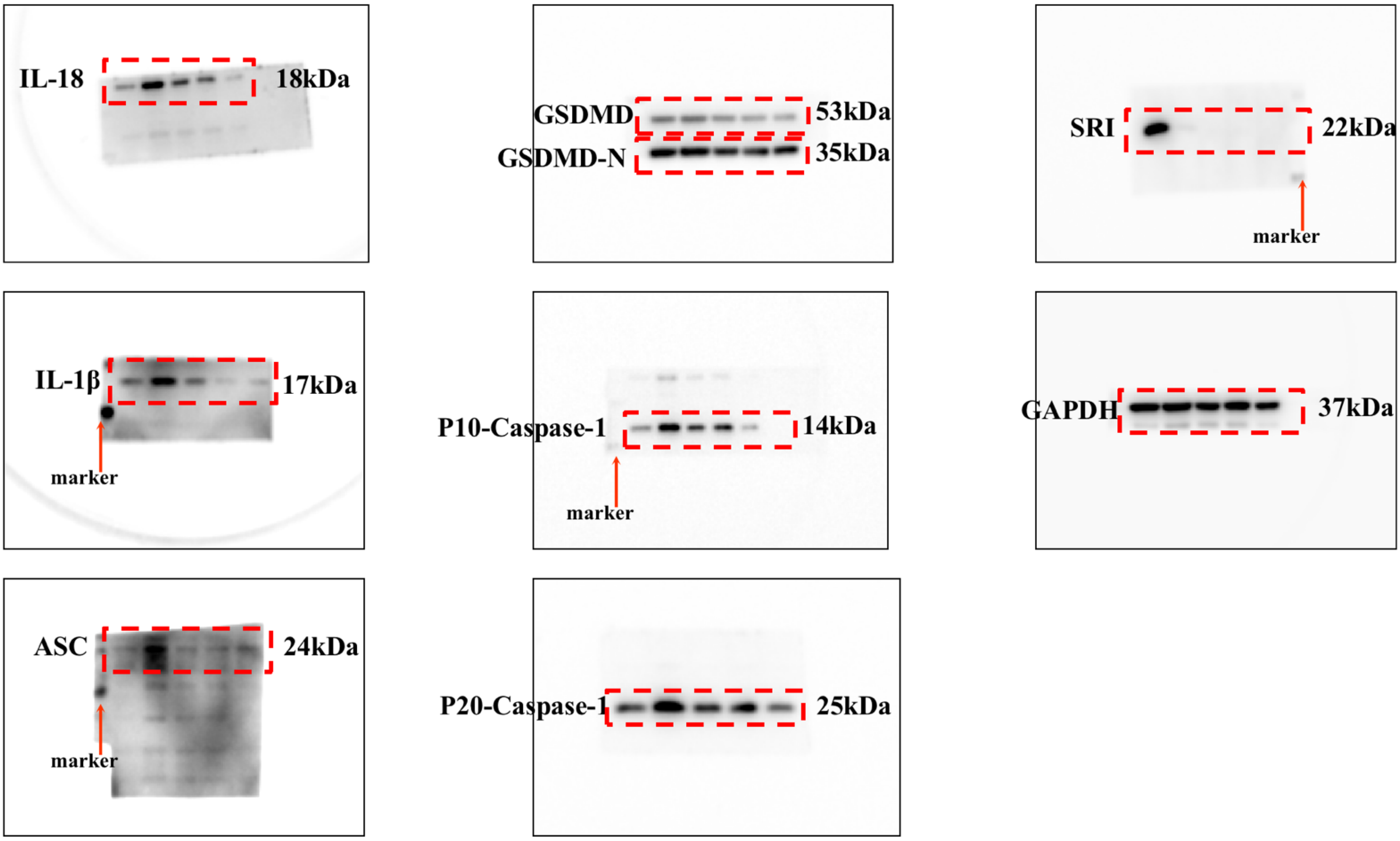
IL-18
18kDa
GSDMD
53kDa
GSDMD-N
35kDa
SRI
22kDa
marker
IL-1β
17kDa
marker
P10-Caspase-1
14kDa
marker
GAPDH
37kDa
ASC
24kDa
marker
P20-Caspase-1
25kDa

Fig.6 C

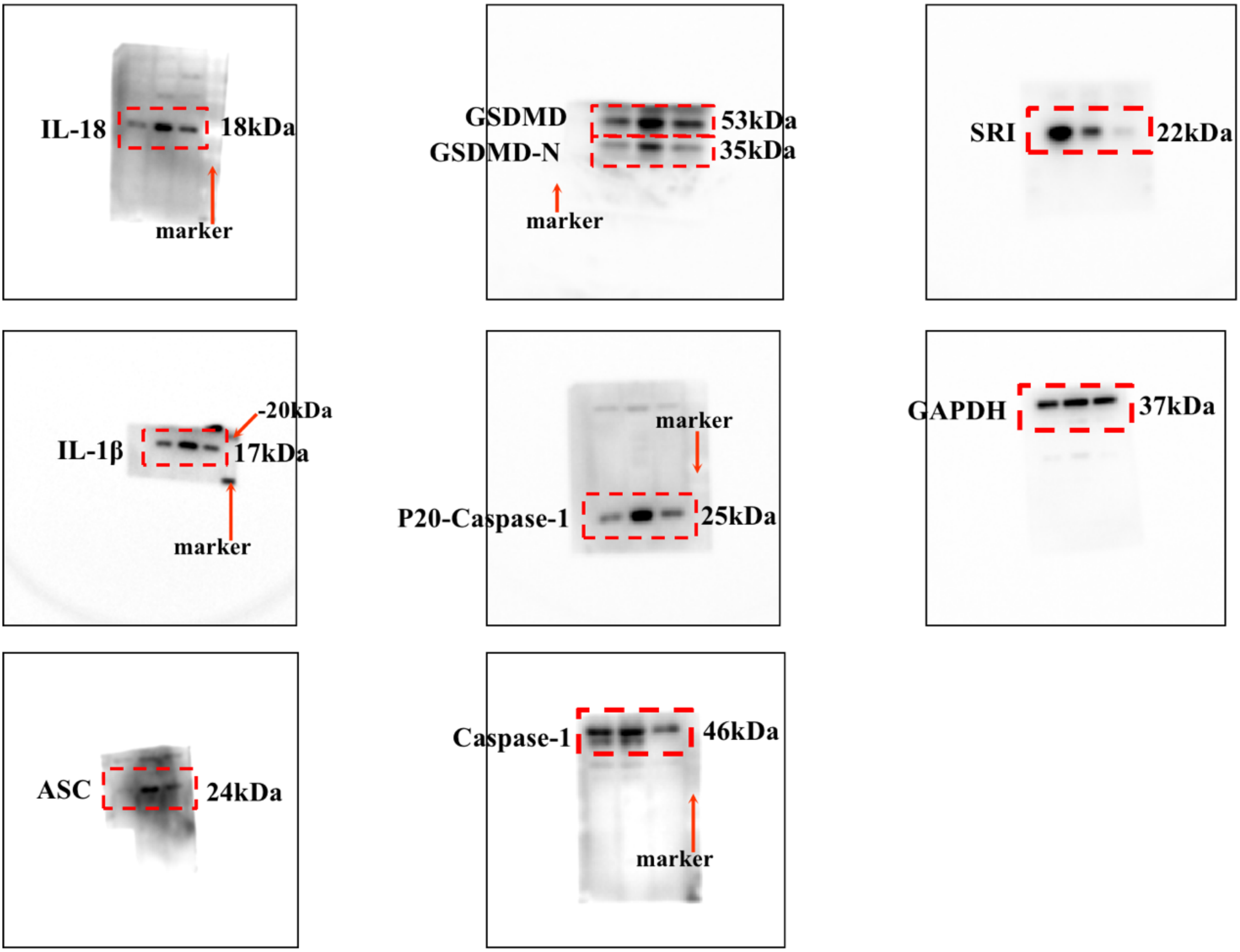
IL-18
18kDa
marker
GSDMD
GSDMD-N
53kDa
35kDa
marker
SRI
22kDa
IL-1β
-20kDa
17kDa
marker
marker
P20-Caspase-1
25kDa
GAPDH
37kDa
ASC
24kDa
Caspase-1
46kDa
marker

Fig.6 D

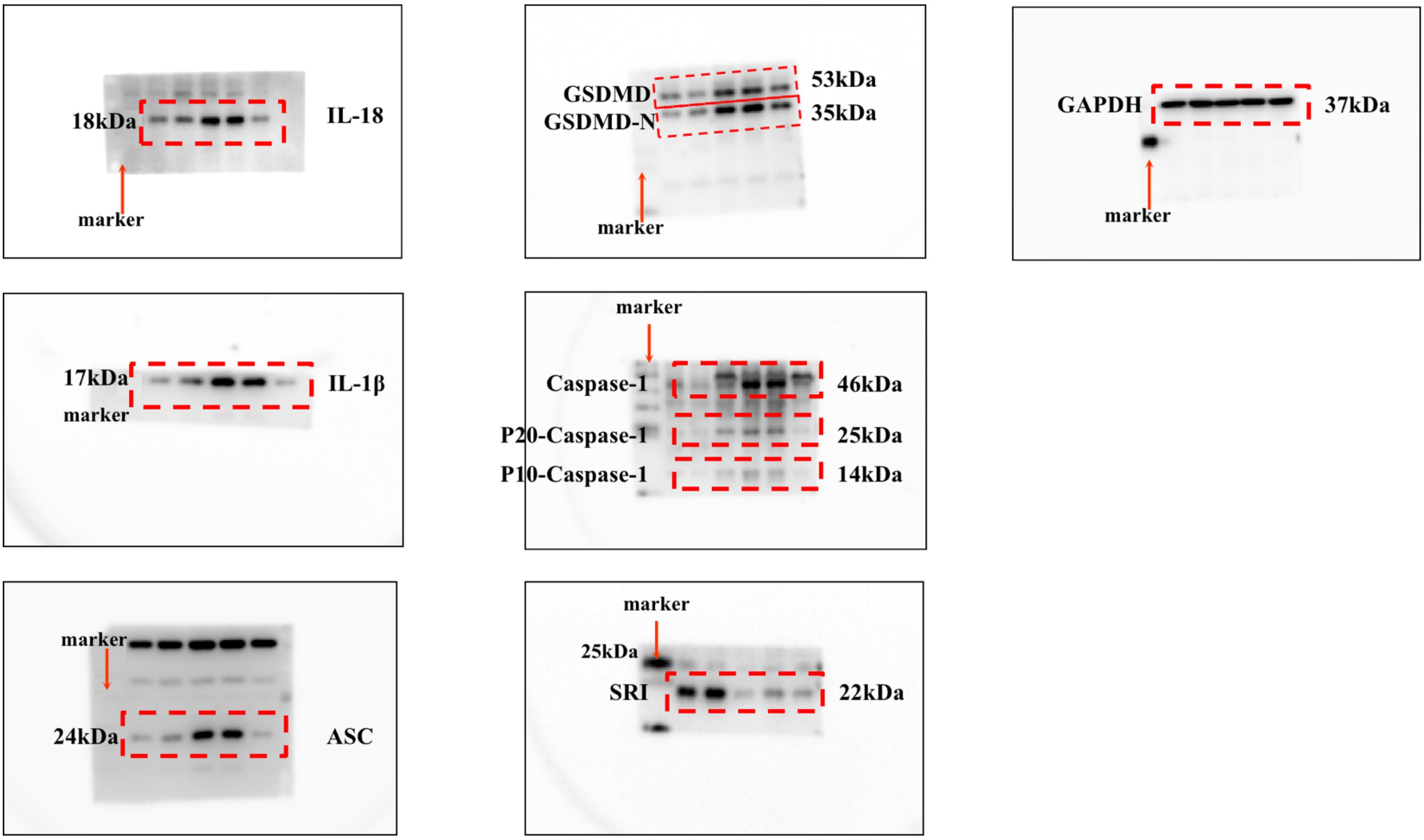

18kDa
IL-18
marker
GSDMD
GSDMD-N
53kDa
35kDa
marker
GAPDH
37kDa
marker
17kDa
IL-1β
marker
marker
Caspase-1
46kDa
P20-Caspase-1
25kDa
P10-Caspase-1
14kDa
marker
24kDa
ASC
marker
25kDa
SRI
22kDa

Fig.S3 A

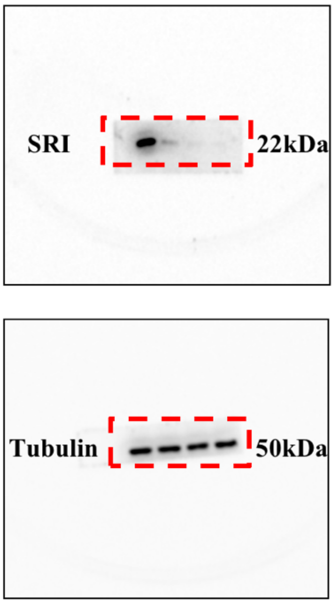

Fig.S3 B

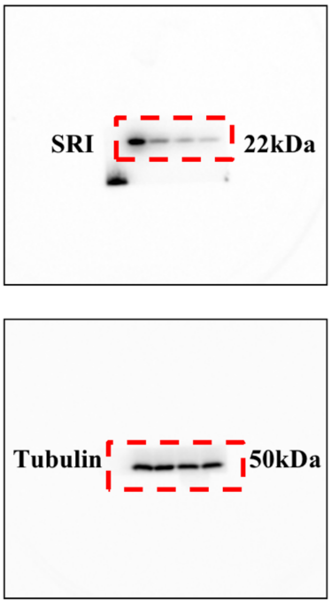

Fig.S3 C

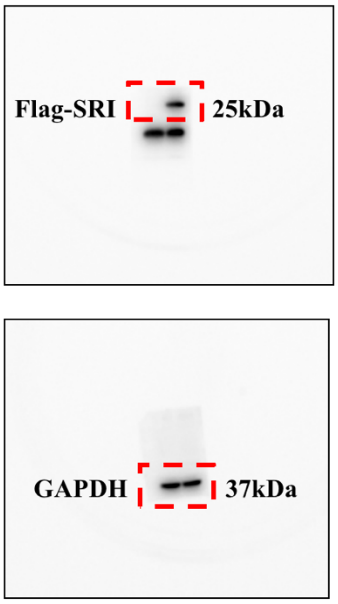

Fig.S3 D

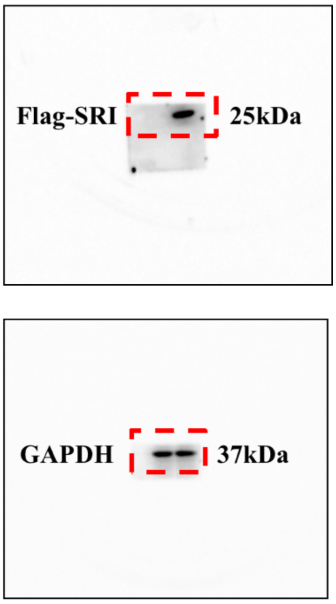

Fig.S5 A

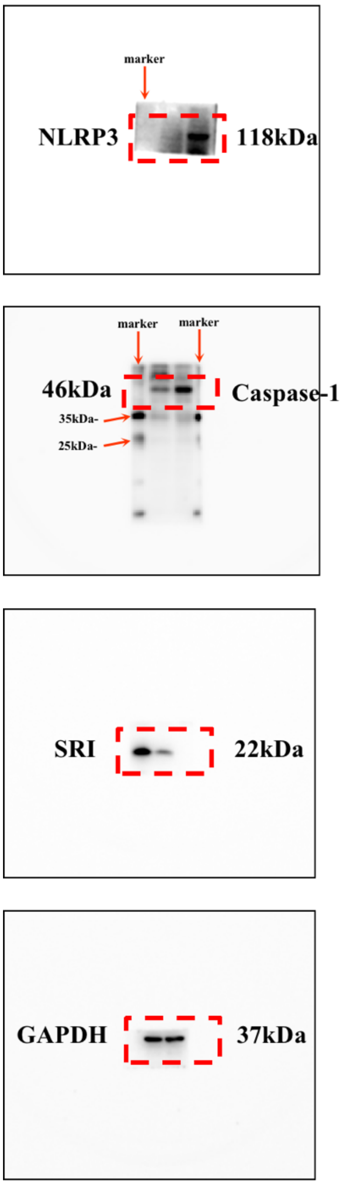

Fig.S5 B

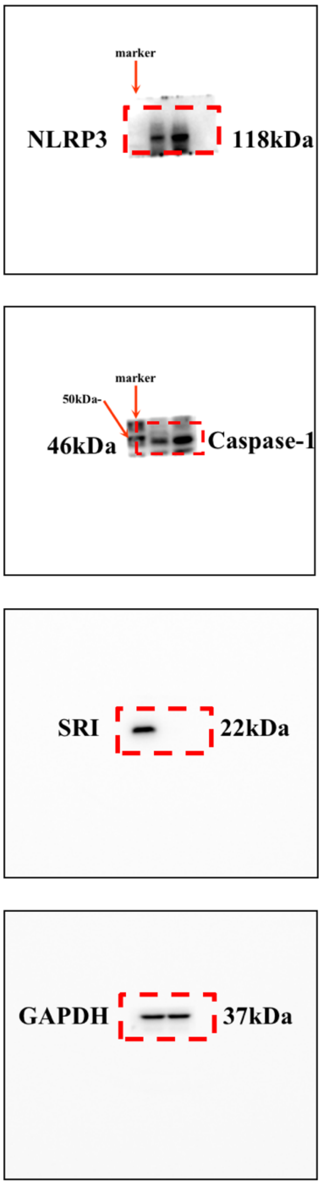

Fig.S5 C

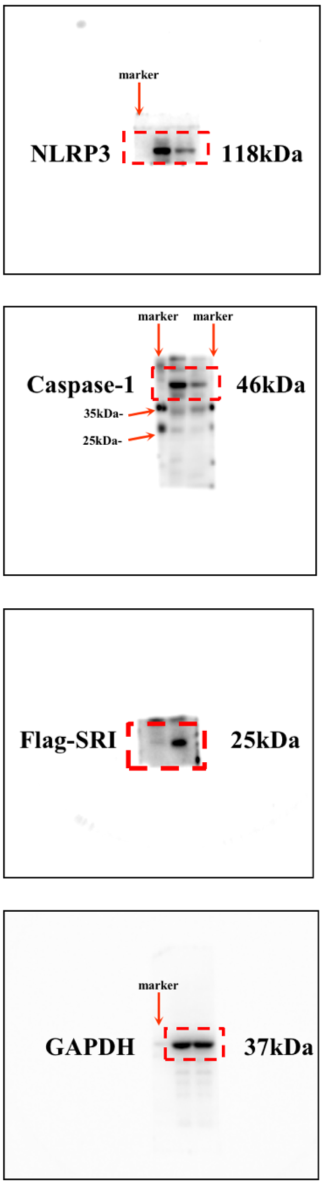

Fig.S5 D

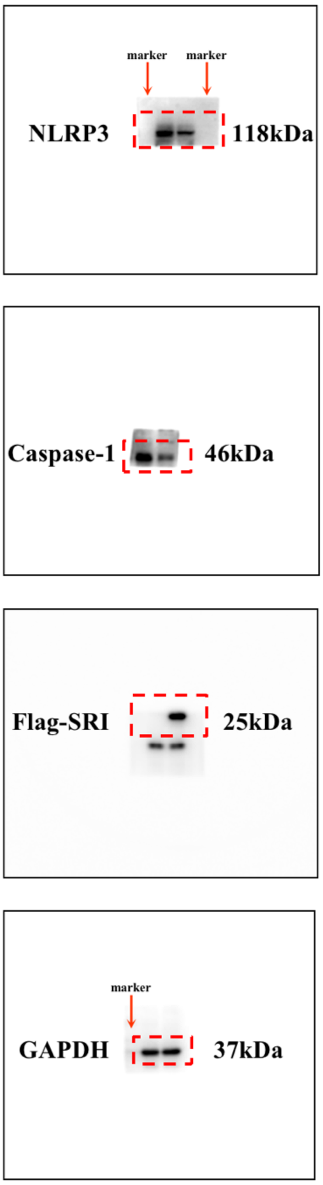

Supplement: Supplementary file 9 — original western blots [file 41419_2023_6096_MOESM9_ESM.pdf]
